# Association of Body Mass Index with DNA Methylation and Gene Expression in Blood Cells and Relations to Cardiometabolic Disease: A Mendelian Randomization Approach

**DOI:** 10.1371/journal.pmed.1002215

**Published:** 2017-01-17

**Authors:** Michael M. Mendelson, Riccardo E. Marioni, Roby Joehanes, Chunyu Liu, Åsa K. Hedman, Stella Aslibekyan, Ellen W. Demerath, Weihua Guan, Degui Zhi, Chen Yao, Tianxiao Huan, Christine Willinger, Brian Chen, Paul Courchesne, Michael Multhaup, Marguerite R. Irvin, Ariella Cohain, Eric E. Schadt, Megan L. Grove, Jan Bressler, Kari North, Johan Sundström, Stefan Gustafsson, Sonia Shah, Allan F. McRae, Sarah E. Harris, Jude Gibson, Paul Redmond, Janie Corley, Lee Murphy, John M. Starr, Erica Kleinbrink, Leonard Lipovich, Peter M. Visscher, Naomi R. Wray, Ronald M. Krauss, Daniele Fallin, Andrew Feinberg, Devin M. Absher, Myriam Fornage, James S. Pankow, Lars Lind, Caroline Fox, Erik Ingelsson, Donna K. Arnett, Eric Boerwinkle, Liming Liang, Daniel Levy, Ian J. Deary

**Affiliations:** 1 Framingham Heart Study, Framingham, Massachusetts, United States of America; 2 Boston University School of Medicine, Boston, Massachusetts, United States of America; 3 Department of Cardiology, Boston Children’s Hospital, Boston, Massachusetts, United States of America; 4 Population Sciences Branch, National Heart, Lung, and Blood Institute, National Institutes of Health, Bethesda, Maryland, United States of America; 5 Centre for Cognitive Ageing and Cognitive Epidemiology, University of Edinburgh, Edinburgh, United Kingdom; 6 Medical Genetics Section, Centre for Genomic and Experimental Medicine, Institute of Genetics and Molecular Medicine, University of Edinburgh, Edinburgh, United Kingdom; 7 Queensland Brain Institute, University of Queensland, Brisbane, Queensland, Australia; 8 Hebrew SeniorLife, Harvard Medical School, Boston, Massachusetts, United States of America; 9 Department of Biostatistics, Boston University, Boston, Massachusetts, United States of America; 10 Molecular Epidemiology and Science for Life Laboratory, Department of Medical Sciences, Uppsala University, Uppsala, Sweden; 11 Department of Epidemiology, School of Public Health, University of Alabama at Birmingham, Birmingham, Alabama, United States of America; 12 Division of Epidemiology and Community Health, School of Public Health, University of Minnesota, Minneapolis, Minnesota, United States of America; 13 Division of Biostatistics, School of Public Health, University of Minnesota, Minneapolis, Minnesota, United States of America; 14 Department of Biostatistics, School of Public Health, University of Alabama at Birmingham, Birmingham, Alabama, United States of America; 15 Center for Epigenetics, Johns Hopkins University School of Medicine, Baltimore, Maryland, United States of America; 16 Icahn Institute for Genomics and Multiscale Biology and Department of Genetics and Genomic Sciences, Icahn School of Medicine at Mount Sinai, New York, New York, United States of America; 17 Human Genetics Center, School of Public Health, University of Texas Health Science Center at Houston, Houston, Texas, United States of America; 18 Department of Epidemiology, University of North Carolina at Chapel Hill, Chapel Hill, North Carolina, United States of America; 19 Cardiovascular Epidemiology, Department of Medical Sciences, Uppsala University, Uppsala, Sweden; 20 Institute for Molecular Bioscience, University of Queensland, Brisbane, Queensland, Australia; 21 Wellcome Trust Clinical Research Facility, Western General Hospital, University of Edinburgh, Edinburgh, United Kingdom; 22 Department of Psychology, University of Edinburgh, Edinburgh, United Kingdom; 23 Alzheimer Scotland Dementia Research Centre, University of Edinburgh, Edinburgh, United Kingdom; 24 Center for Molecular Medicine and Genetics and Department of Neurology, Wayne State University, Detroit, Michigan, United States of America; 25 Children’s Hospital Oakland Research Institute, Oakland, California, United States of America; 26 HudsonAlpha Institute for Biotechnology, Huntsville, Alabama, United States of America; 27 Human Genome Sequencing Center, Baylor College of Medicine, Houston, Texas, United States of America; 28 Department of Medicine, Division of Cardiovascular Medicine, Stanford University School of Medicine, Stanford, California, United States of America; 29 Departments of Epidemiology and Biostatistics, School of Public Health, Harvard University, Boston, Massachusetts, United States of America; 30 Brown Foundation Institute of Molecular Medicine, University of Texas, Houston, Texas, United States of America; 31 College of Public Health, University of Kentucky, Lexington, Kentucky, United States of America; Kings College London, UNITED KINGDOM

## Abstract

**Background:**

The link between DNA methylation, obesity, and adiposity-related diseases in the general population remains uncertain.

**Methods and Findings:**

We conducted an association study of body mass index (BMI) and differential methylation for over 400,000 CpGs assayed by microarray in whole-blood-derived DNA from 3,743 participants in the Framingham Heart Study and the Lothian Birth Cohorts, with independent replication in three external cohorts of 4,055 participants. We examined variations in whole blood gene expression and conducted Mendelian randomization analyses to investigate the functional and clinical relevance of the findings. We identified novel and previously reported BMI-related differential methylation at 83 CpGs that replicated across cohorts; BMI-related differential methylation was associated with concurrent changes in the expression of genes in lipid metabolism pathways. Genetic instrumental variable analysis of alterations in methylation at one of the 83 replicated CpGs, cg11024682 (intronic to *sterol regulatory element binding transcription factor 1* [*SREBF1*]), demonstrated links to BMI, adiposity-related traits, and coronary artery disease. Independent genetic instruments for expression of *SREBF1* supported the findings linking methylation to adiposity and cardiometabolic disease. Methylation at a substantial proportion (16 of 83) of the identified loci was found to be secondary to differences in BMI. However, the cross-sectional nature of the data limits definitive causal determination.

**Conclusions:**

We present robust associations of BMI with differential DNA methylation at numerous loci in blood cells. BMI-related DNA methylation and gene expression provide mechanistic insights into the relationship between DNA methylation, obesity, and adiposity-related diseases.

## Abstract 2

Daniel Levy and colleagues examine mechanisms that could link body mass index to cardiovascular diseases via gene expression.

## Author Summary

**Why Was This Study Done?:**

- Genetic sequence variants explain only a modest proportion of the variation in body mass index (BMI) and cardiometabolic disease in the general population.
- There is limited understanding of the link of DNA methylation—a well-characterized epigenetic modification—with BMI and cardiometabolic disease in the general population.

**What Did the Researchers Do and Find?:**

- We conducted a cross-sectional analysis of the association of BMI with leukocyte DNA methylation at over 400,000 sites in the genome among 7,798 community-dwelling adults.
- We identified associations between BMI and methylation at 83 replicated sites (including 50 novel sites) and concurrent differences in expression in whole blood of genes overrepresented in lipid metabolism pathways.
- Using genetic sequence variants to model exposure to differential DNA methylation and tissue-specific gene expression, we found differential methylation and expression of *SREBF1* to be implicated in BMI, adiposity-related traits, and coronary artery disease.
- Using genetic sequence variants to model exposure to differences in BMI, we found a substantial proportion of the differentially methylated sites (16 of 83) to be downstream of BMI.

**What Do These Findings Mean?:**

- Evidence is accumulating that epigenetic modifications, such as DNA methylation, are related to obesity-related diseases in the general population.
- We provide support for a role of genomic regulation of a lipid metabolism transcription factor, *SREBF1*, in adiposity and coronary artery disease.
- Mendelian randomization approaches can help prioritize relevant loci for future functional studies, but the cross-sectional observational nature of our study limits definitive causal inference.

## Introduction

Obesity is highly prevalent in developed nations [1] and contributes to a substantial burden of morbidity and mortality [2,3]. Despite advances in the understanding of genetic variants, lifestyle factors, and gene–environment interactions associated with obesity [4–7], much of the interindividual variation in body weight remains unexplained by measurable lifestyle and genetic factors. DNA methylation, one of the most frequent and well-characterized epigenetic modifications, reflects at the molecular level a wide range of environmental exposures and genetic influences [8]. By stabilizing chromatin structure and altering gene expression, DNA methylation has the potential to affect an individual’s susceptibility to obesity (see review in [9]). Further, changes in the methylation of DNA may occur secondarily to obesity and may consequently influence the development of adiposity-related diseases such as diabetes, dyslipidemia, hypertension, and cardiovascular disease. Large gaps in knowledge remain as to how human epigenetic modifications relate to obesity and its sequelae.

Epigenetic biomarkers represent a largely untapped precision medicine resource to guide therapy decisions using an individual’s epigenetic profile obtained from blood samples [10]. Identification of clinically relevant epigenetic loci in blood holds the potential to create a foundation upon which to base future functional studies and trials to test epigenetically guided clinical decision making for cardiometabolic diseases. In addition, we may gain novel insights into the molecular underpinnings of obesity and adiposity-related diseases through the study of differentially methylated DNA loci in blood. Doing so may lead to the identification of biologically relevant therapeutic targets.

The present study provides results of an epigenome-wide association study (EWAS) of body mass index (BMI) in over 3,700 participants from the Framingham Heart Study (FHS) and the Lothian Birth Cohorts (LBCs) of 1921 and 1936 (LBC1921 and LBC1936). We conducted independent external replication in over 4,000 individuals from the Atherosclerosis Risk in Communities (ARIC), Genetics of Lipid Lowering Drugs and Diet Network (GOLDN), and Prospective Investigation of the Vasculature in Uppsala Seniors (PIVUS) cohort studies. We examined the functional relevance of the identified loci by interrogating the known trans-tissue regulatory functions and concomitant changes in gene expression in blood. In addition, we explored the clinical relevance of the findings for adiposity-related diseases with genetic instrumental variable (IV) analyses using bidirectional and two-step trans-tissue Mendelian randomization (MR) approaches [11–13].

## Methods

### Study Design

**Fig 1:**
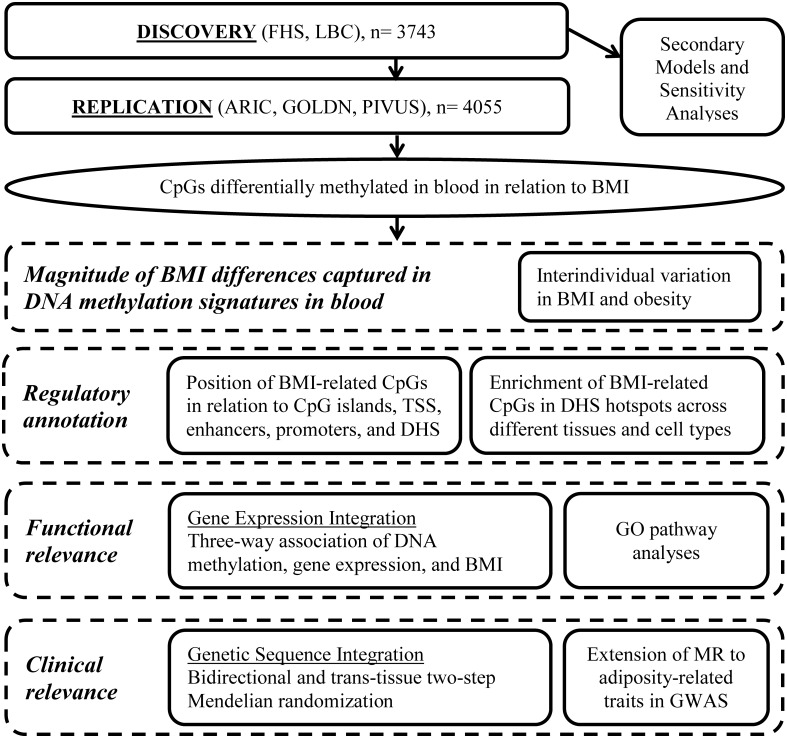
Series of analyses conducted for the epigenome-wide association study of body mass index. ARIC, Atherosclerosis Risk in Communities; BMI, body mass index; DHS, DNase I hypersensitive site; FHS, Framingham Heart Study; GO, Gene Ontology; GOLDN, Genetics of Lipid Lowering Drugs and Diet Network; GWAS, genome-wide association study; LBC, Lothian Birth Cohorts; MR, Mendelian randomization; PIVUS, Prospective Investigation of the Vasculature in Uppsala Seniors; TSS, transcription start site.

The study includes two major components. First, we conducted an EWAS of BMI. Second, BMI-related differentially methylated loci were taken forward for further analyses to better understand the magnitude of association, regulatory annotation, functional implications, and clinical relevance (Fig 1). The discovery/replication design and secondary models for the BMI EWAS were defined a priori (S1 Text). Downstream analyses to characterize the discovered loci were outlined a priori, but the final approach was primarily driven by the findings and concurrent advancements in the field.

### Ethics

The FHS protocols and participant consent forms were approved by the institutional review board of Boston University School of Medicine. Ethics permission for the LBC1921 was obtained from the Lothian Research Ethics Committee (Wave 1: LREC/1998/4/183). Ethics permission for the LBC1936 was obtained from the Multi-Centre Research Ethics Committee for Scotland (Wave 1: MREC/01/0/56) and the Lothian Research Ethics Committee (Wave 1: LREC/2003/2/29). Written informed consent was obtained from all discovery cohort (FHS and LBC) and replication cohort (ARIC, GOLDN, and PIVUS) participants.

### Study Participants

Data for the discovery phase of this investigation were drawn from the FHS offspring cohort [14] and the LBCs of 1921 and 1936 [15–17]. As previously described [14], the FHS offspring cohort was initially recruited in 1971 and included 5,124 offspring (and their spouses) from the FHS original cohort [18]. The eligible sample for this investigation was from the 3,021 participants in the FHS offspring cohort who attended the eighth examination cycle from 2005 to 2008. The LBC1921 and LBC1936 samples derive from the Scottish Mental Surveys of 1932 and 1947, respectively, when nearly all 11-y-old children in Scotland completed an IQ-type test in school. The LBC studies provided follow-up of surviving participants, most of whom were living in the Lothian region (Edinburgh city and outskirts) of Scotland. The current study draws upon the older-age baseline examinations of 551 participants in LBC1921 recruited in 1999–2001 and 1,091 participants in LBC1936 recruited in 2004–2007.

### Anthropometric Measurements

Height and weight were measured in each study using established protocols as described in detail in the S1 Methods. BMI was calculated as weight (in kilograms) divided by height (in meters) squared.

### Molecular Genomics

DNA from whole blood samples was collected at the same examination assessment as the anthropometric and covariate measurements in both studies. DNA methylation, assayed with the Infinium HumanMethylation450 BeadChip [19] (Illumina), was available for 2,846 FHS participants and 1,518 LBC participants (514 from LBC1921 and 1,004 from LBC1936). Details of rigorous quality control, normalization procedures, and exclusions of non-autosomal probes, cross-hybridizing probes, and probes with underlying single nucleotide polymorphisms (SNPs) are described in S1 Methods. Each discovery and replication cohort conducted cohort-specific preprocessing pipelines that allowed each cohort to address study-specific technical and batch effects. This design allowed for the selection of true biological signals independent of bias introduced from uniform processing methods. After quality control in the discovery cohorts, there were 402,358 shared CpG (cytosine-phosphate-guanine) methylation probes available for analyses in 2,377 FHS and 1,366 LBC participants (446 from LBC1921 and 920 from LBC1936). Final sample size was determined by the number of community-based participants in the discovery cohorts who consented to genomic studies and who had available DNA and methylation assays passing quality control measures. In the FHS, SNP data were obtained from the Affymetrix 550K Array imputed to the 1000 Genomes Project reference panel, as previously reported [20]. The LBC samples were genotyped using the Illumina Human610-Quad v1.0 genotyping platform and imputed to the 1000 Genomes Project reference panel as well. Gene expression in blood was available in the FHS and was measured using the Affymetrix Human Exon 1.0 ST GeneChip as described in S1 Methods.

### Epigenome-Wide Association Study of BMI

In the FHS, linear mixed effects regression models were conducted to test the association between site-specific DNA methylation and BMI. The primary model was adjusted for age, sex, family relatedness (random effect), and surrogate variables (to account for differential cell proportions and technical effects) [21], with BMI as the independent variable of interest and DNA methylation (inverse-normal transformed) as the dependent variable. In the LBC, linear regression models were conducted adjusting for age, sex, and white blood cell counts, with each DNA methylation probe (residual taken forward from a generalized linear model with a logistic link function adjusting for technical and batch effects) as the dependent variable and BMI as the independent variable of interest. Further analytical details for the discovery cohorts are described in S1 Methods. In both cohorts, secondary models were conducted: (1) additionally adjusting for smoking status, (2) restricted to participants with BMI 18–35 kg/m^2^ in order to avoid confounding due to frailty or morbid obesity and obesity-related diseases, and (3) testing for age and sex interactions. Results from the FHS and LBCs were meta-analyzed using methods that weighted the *p-*value by sample size [22]. Directional consistency of statistically significant cohort-specific effects was confirmed for all methylome-wide significant findings from the discovery meta-analysis. We focused our analyses on the resultant test statistic and direction of effect from the independent variable of interest (BMI) as the cohort-specific linear regression coefficients were not directly comparable due to the differences in the preprocessing approach between cohorts. The threshold for statistical significance in the discovery phase was defined by Bonferroni correction for multiple testing to be 0.05/405,000 (*p-*value < 1.2 × 10^−7^). A flowchart of analyses conducted is presented in Fig 1.

### External Replication of EWAS Findings

The methylome-wide significant CpGs from the FHS and LBC meta-analysis were taken forward to external replication in three independent cohorts that used the same methylation microarray: the ARIC study, using whole-blood-derived DNA from 2,096 participants of African ancestry; the GOLDN study, using DNA derived from CD4+ cells from 992 participants of European ancestry; and the PIVUS study, using whole blood-derived DNA from 967 participants of Swedish ancestry. Description and analytical methods of the replication cohorts are supplied in S1 Methods. Replication cohorts also conducted cohort-specific preprocessing. Replication was examined within each cohort individually and then in a meta-analysis of all three replication cohorts (using *p-*value-weighted methods and ensuring directional consistency as described above). The threshold for statistically significant replication was determined by Bonferroni correction to be 0.05 divided by the number of CpGs taken forward from discovery.

### Sensitivity Models Adjusting EWAS Findings for Potential Confounding by Genetic Variation

In order to demonstrate whether the DNA methylation and BMI association results were independent of genetic variants influencing methylation (methylation quantitative trait loci [meQTLs]), we conducted sensitivity models in the FHS for the replicated BMI-related CpGs conditional on the top cis-meQTL (selected by lowest *p-*value; ±500 kb from the CpG) for each replicated CpG. The approach to identify cis-meQTLs for the BMI-related CpGs is described in S1 Methods.

### Interindividual Variation in BMI and Distribution of Obesity in Relation to EWAS Findings

In order to determine the magnitude of variation in BMI contained within the studied epigenetic signatures in blood, we examined the variation captured in three ways. First, we examined the increase in model *R*^2^ starting from the baseline covariate-only linear regression model, with BMI as the dependent variable, when adding nonredundant (|*r*| < 0.7) replicated CpGs as independent variables in order of decreasing statistical significance. We conducted this analysis in two discovery test sets: (1) methylome-wide significant CpGs in the FHS only were tested in the LBCs and (2) replicated nonredundant CpGs from the BMI EWAS were tested in one of the replication cohorts, PIVUS. Due to differences from the discovery cohorts in ethnicity (African ancestry in ARIC) and cell line (CD4+ cells in GOLDN), we conducted the variation analyses only in PIVUS. Second, we created an additive composite measure of the same nonredundant statistically significant replicated CpGs weighted by effect size. The composite methylation measure was generated for each individual by summing the product of the methylation beta-value and the cohort-specific effect size (including direction of effect) for each of the nonredundant replicated CpGs. The distribution of BMI and prevalence of obesity (BMI ≥ 30 kg/m^2^) was assessed across deciles of the additive weighted composite measure in the PIVUS cohort. Third, the change in BMI and odds of overweight (BMI 25–29.9 kg/m^2^) and obesity were tested in age- and sex-adjusted linear and logistic regression models for each standard deviation (SD) change in the additive weighted composite measure in the PIVUS cohort. The weighted summation of the composite methylation measure was converted to SD units (mean = 0, SD = 1) to enhance interpretability of results. As some of the cross-sectional differential methylation changes were expected to be secondary to BMI differences, the purpose of these analyses was not to develop a biomarker or risk predictor for cross-sectional BMI measures but to determine if a large proportion of variation in BMI and obesity, and hence obesity-related cardiometabolic risk, is reflected in the blood DNA methylation patterns. Further analyses examine the molecular pathways that are affected and attempt to infer which methylation changes are causally influencing BMI, which are secondary to BMI differences, and which have relevance for clinical disease outcomes.

### Gene Expression Analyses

We analyzed whole blood gene expression data in the FHS to identify which BMI-related differentially methylated CpGs demonstrated association with altered gene expression. The replicated CpGs were tested using linear mixed effects models for association, with the expression level of the corresponding gene in whole blood (based on annotation by the manufacturer) as the dependent variable and DNA methylation as the independent variable, adjusted for age, sex, and technical and batch effects (further details in S1 Methods).

### Functional and Regulatory Annotation

We studied the Gene Ontology (GO) biological process, molecular function, and cellular component pathways (release 2016-08-22) of the genes identified in the BMI EWAS using the PANTHER (protein annotation through evolutionary relationship) overrepresentation test [23]. Secondarily, we restricted analysis to the higher certainty genes shown to have altered whole blood gene expression in association with BMI-related differential methylation, as described in the previous section. If multiple probes were annotated to the same gene, then the gene was included only once (unweighted). As the methylation array covers 99% of RefSeq genes, the background universe of genes tested was not restricted. Results were corrected for multiple testing within each category.

In addition, we used eFORGE v1.2 (http://eforge.cs.ucl.ac.uk/) [24] to identify if the replicated CpGs were enriched in DNase I hypersensitive sites (DHSs) (markers of active regulatory regions) and loci with overlapping histone modifications (H3Kme1, H3Kme4, H3K9me3, H3K27me3, and H3K36me3) across available cell lines and tissues from Roadmap Epigenomics Project, BLUEPRINT Epigenome, and ENCODE (Encyclopedia of DNA Elements) consortia data [25–27].

### Bidirectional and Two-Step Trans-tissue Mendelian Randomization

IV analyses using SNPs as IVs for (1) DNA methylation, (2) gene expression, and (3) BMI were conducted in order to infer potential causal relationships between EWAS findings, BMI, and adiposity-related diseases (the series of analyses conducted is outlined in Table 1). The detailed approach is provided in S1 Methods. In brief, differences in methylation and expression were modeled using quantitative trait loci (QTLs), thus leveraging the contribution of genetic variation to epigenetic traits to infer causal relations. Blood QTL IVs were selected as the single top SNP methylation or expression association (by lowest *p-*value) in the FHS with replication in the external cohorts or public datasets. As QTLs vary in effect in different tissue types, we selected tissue-specific methylation and expression QTLs to examine tissue-specific effects (details in S1 Methods). To model the effect of BMI on methylation (reverse causation), the IV for BMI was assembled as an additive weighted genetic risk score from the 97 genome-wide significant SNPs from the Genetic Investigation of ANthropometric Traits (GIANT) consortium 2015 genome-wide association study (GWAS) results [7]. A sensitivity analysis utilizing a single SNP in the *FTO* (*fat mass and obesity associated*) locus as the IV for BMI was conducted to examine an IV less prone to pleiotropy bias but also less powerful to detect potential causal relations.

**Table 1:** Schema of instrumental variable analyses conducted in order to infer the potential causal relations between DNA methylation, gene expression, BMI, and adiposity-related disease.

Method	Exposure	IV	Source of IV	Selection	Outcome	Setting
Forward MR	DNA methylation	meQTL	FHS/replication cohorts	All replicated CpGs (as the exposure)	BMI	FHS/GIANT consortium
Two-step MR—first step	DNA methylation	meQTL	FHS/replication cohorts	Significant in forward MR	Gene expression in multiple tissues	FHS/external eQTL datasets from blood, liver, and adipose tissue
Two-step MR—second step	Gene expression in multiple tissues	eQTL	FHS/GTEx/external eQTL datasets	Significant in forward MR	BMI	FHS/GIANT consortium
Extension of causal relations to adiposity-related traits	DNA methylation	meQTL	FHS/replication cohorts	Significant in forward MR	Adiposity-related traits	GWAS results
	Gene expression in multiple tissues	eQTL	FHS/GTEx/external eQTL datasets	Significant in two-step MR	Adiposity-related traits	GWAS results
Reverse MR	BMI	BMI GRS	GWAS results	All replicated CpGs (as the outcome)	DNA methylation	FHS

BMI, body mass index; eQTL, expression quantitative trait locus; FHS, Framingham Heart Study; GIANT, Genetic Investigation of ANthropometric Traits; GRS, genetic risk score; GTEx, Genotype-Tissue Expression Project; GWAS, genome-wide association study; IV, instrumental variable; meQTL, methylation quantitative trait locus; MR, Mendelian randomization.

Forward MR, using the two-stage least squares method, tests the causal relation of differential methylation with BMI. SNP IVs that implicated a causal effect of differential methylation on BMI from the forward MR (Bonferroni-corrected and, secondarily, nominal causal *p-*value < 0.05) were tested in the trans-tissue two-step MR. The trans-tissue two-step MR was implemented to further break down the relationship between DNA methylation and BMI and to infer whether the hypothesized mediator (gene expression in multiple tissues) is influenced by the exposure (DNA methylation) and, second, whether the mediator (gene expression in multiple tissues) affects the outcome (BMI). SNP IVs that implicated a causal effect of differential methylation and expression on BMI were tested for associations with adiposity-related phenotypes from published GWAS results. Finally, the reverse MR was conducted to test the causal relation of BMI with downstream changes in DNA methylation.

## Results

### Discovery Cohort Characteristics

The discovery sample included 3,743 individuals: 2,377 from the FHS and 1,366 from the LBCs (*n =* 446 from LBC1921 and *n =* 920 from LBC1936). The FHS, LBC1921, and LBC1936 cohorts were older adults (mean [SD] age 67 [9], 79 [1], and 70 [1] y, respectively) and had similar sex distribution (50%–60% female) and proportion of current smokers (8%–11%) (Table 2).

**Table 2:** Study characteristics of the Framingham Heart Study and Lothian Birth Cohort participants (discovery cohorts) at the time of DNA methylation assays.

Characteristic	FHS	LBC1936	LBC1921
***N***	2,377	920	446
**Age (years)**	67 ± 9	70 ± 1	79 ± 1
**Female**	55%	40%	61%
**BMI (kg/m**^**2**^**)**	28.3 ± 5.4	27.8 ± 4.4	26.2 ± 4.0
**Current smoking**	8%	11%	7%

Data are counts, means ± standard deviation, and proportions as appropriate.

BMI, body mass index; FHS, Framingham Heart Study; LBC, Lothian Birth Cohort.

### Epigenome-Wide Association Study of BMI

#### Discovery

In the FHS-LBC EWAS meta-analysis, 135 CpGs were significantly associated with BMI after correction for multiple testing in the primary age- and sex-adjusted model (*p* < 1.2 × 10^−7^; full list and regression coefficients are provided in S1 Table; Q-Q plots in S1 and S2 Figs; Manhattan plot in S3 Fig; genomic inflation factor of discovery meta-analysis, λ = 1.14). Similar results were observed following additional adjustment for smoking status and after excluding 313 individuals with BMI outside of 18–35 kg/m^2^ (Models 2–3 in S2 Table; S4 Fig).

#### External replication

The 135 statistically significant CpGs from the discovery BMI EWAS meta-analysis (primary model) were tested for external replication in the ARIC (*n =* 2,096), GOLDN (*n =* 992), and PIVUS (*n =* 967) cohorts. There was external replication of 83 of 135 CpGs in at least one cohort (73 in ARIC, 22 in GOLDN, and 19 in PIVUS; S5 Fig) at *p-*value < 3.7 × 10^−4^ (Bonferroni-corrected *p-*value for 135 tests), and 83 of 135 CpGs replicated in the meta-analyses of the three replication cohorts and were taken forward for subsequent analyses (S3 Table). Greater methylation was associated with higher BMI at 49 (59%) of the 83 replicated CpGs. The majority of BMI-related CpGs (65%–85% of CpGs depending on the cohort) had mean sample CpG methylation levels between 20% and 80% (S4 Table). Fifty of the 83 replicated differentially methylated CpGs have not been previously reported in microarray-based EWASs of BMI [28–36] (Table 3).

**Table 3:** Fifty novel replicated differentially methylated CpGs associated with BMI sorted by *p-*value in the discovery cohorts.

CpG	Gene	Discovery		Replication						
		FHS-LBC (*n =* 3,743)		ARIC (*n =* 2,096)		GOLDN (*n =* 992)		PIVUS (*n =* 967)		Meta-analysis
		*p*-Value	Dir	*p*-Value	Dir	*p*-Value	Dir	*p*-Value	Dir	*p*-Value
cg17501210	*RPS6KA2*	4.14E−21	− − − −	1.72E−04	−	1.36E−06	−	4.15E−02	−	1.07E−06
cg14870271	*LGALS3BP*	4.87E−16	+ + + +	2.28E−04	+	3.62E−03	+	4.80E−02	+	1.51E−05
cg11202345	*LGALS3BP*	6.22E−15	+ + + +	7.70E−05	+	1.36E−02	+	1.83E−01	+	4.20E−05
cg19750657	*UFM1*	2.21E−13	+ + + +	5.55E−06	+	6.92E−07	+	2.27E−08	+	5.60E−13
cg17901584	*DHCR24*	2.87E−12	− − − −	6.26E−07	−	1.23E−04	−	7.99E−07	−	4.14E−12
cg10179300	*TRIO*	5.71E−12	+ + + +	7.37E−04	+	1.16E−02	+	8.82E−02	+	1.06E−04
cg10508317	*SOCS3*	2.90E−11	− − − −	1.31E−03	−	3.49E−05	−	2.85E−02	−	1.12E−05
cg17836612	*LGALS3BP*	1.05E−10	+ + + +	4.73E−06	+	1.99E−03	+	7.40E−04	+	1.24E−08
cg26950531	*DPF1*	1.13E−10	− − − −	6.28E−04	−	6.49E−03	−	9.90E−04	−	2.22E−06
cg21429551	*GARS*	1.31E−10	− − − −	3.95E−03	−	1.13E−05	−	8.58E−02	−	6.76E−05
cg01881899	*ABCG1*	1.37E−10	+ + + +	4.90E−03	+	4.99E−04	+	8.25E−02	+	1.98E−04
cg17782974	*TRIM8*	1.79E−10	+ + + +	7.54E−06	+	5.71E−02	+	1.39E−01	+	1.05E−05
cg16611584	*AKAP10*	2.40E−10	+ + + +	2.08E−04	+	9.92E−01	+	1.98E−02	+	1.40E−04
cg07730360		3.32E−10	+ + + +	1.37E−06	+	3.31E−02	+	5.38E−02	+	8.00E−07
cg17738521	*HIVEP2*	3.51E−10	− − − −	2.36E−04	−	1.37E−01	−	7.56E−02	−	1.07E−04
cg18098839	*GOLIM4*	1.21E−09	− − − +	1.48E−03	−	1.12E−03	−	3.66E−04	−	1.42E−06
cg00108715	*NT5DC2*	1.71E−09	+ + + +	1.98E−07	+	4.60E−01	+	4.00E−04	+	1.61E−08
cg24531955	*LOXL2*	2.26E−09	− − − −	3.85E−05	−	1.67E−02	−	9.20E−03	−	1.46E−06
cg17058475	*CPT1A*	2.28E−09	− − − −	3.18E−04	−	1.64E−05	−	1.29E−01	−	1.17E−05
cg24678869	*DENND4B*	2.56E−09	+ + + +	4.72E−08	+	6.92E−01	−	4.46E−01	+	2.64E−05
cg13134297		3.21E−09	− − − −	1.10E−04	−	5.62E−03	−	2.06E−01	−	4.59E−05
cg10474597	*SERPINE3*	3.82E−09	+ + + +	3.51E−04	+	2.13E−01	+	1.39E−04	+	2.19E−06
cg03725309	*SARS*	3.93E−09	− − − −	2.67E−04	−	2.75E−04	−	2.43E−03	−	6.00E−07
cg26894079	*ASAM*	4.06E−09	− − − −	2.31E−06	−	4.60E−05	−	1.96E−03	−	3.52E−09
cg22012981	*ACOX2*	4.65E−09	+ + + +	2.64E−04	+	2.27E−02	+	1.97E−03	+	2.71E−06
cg26361535	*ZC3H3*	5.46E−09	− + + +	1.56E−07	+	2.65E−02	+	1.37E−02	+	3.43E−08
cg04286697	*B3GNT7*	5.96E−09	+ + + +	8.70E−06	+	4.51E−02	+	7.53E−01	+	1.22E−04
cg26651978		6.24E−09	− − − −	6.42E−03	−	3.07E−04	−	2.76E−03	−	1.68E−05
cg19017142		7.86E−09	− − − −	1.48E−04	−	1.92E−02	−	1.80E−02	−	8.35E−06
cg10919522	*C14orf43*	9.71E−09	− − − −	3.98E−06	−	2.78E−04	−	8.58E−04	−	5.44E−09
cg25649826	*USP22*	1.05E−08	+ + + +	9.69E−06	+	1.41E−03	+	6.62E−02	+	1.18E−06
cg07037944	*DAPK2*	1.19E−08	− − − −	2.47E−04	−	1.36E−01	−	4.12E−09	−	4.32E−09
cg24145109		1.23E−08	+ + + +	2.69E−05	+	8.59E−02	+	2.28E−04	+	1.62E−07
cg01751802	*KANK2*	1.32E−08	+ + + +	6.33E−07	+	8.89E−01	+	3.31E−01	+	4.30E−05
cg13274938	*RARA*	1.43E−08	+ + + +	5.48E−07	+	3.47E−03	+	3.70E−02	+	9.57E−08
cg11673687	*SLC9A1*	2.52E−08	+ + + +	7.80E−04	+	8.37E−01	−	7.96E−03	+	2.51E−04
cg26800893	*ATPGD1*	2.87E−08	− − − −	5.04E−04	−	8.10E−04	−	3.80E−04	−	4.29E−07
cg01368219	*CACNA2D3*	3.20E−08	+ + + +	3.90E−06	+	2.21E−02	+	2.62E−02	+	6.87E−07
cg01130991		4.00E−08	+ + + +	7.46E−06	+	2.49E−04	+	2.91E−02	+	2.19E−07
cg27470213	*LGALS3BP*	4.63E−08	− − − −	3.88E−05	−	1.23E−03	−	4.18E−03	−	2.64E−07
cg26955383	*CALHM1*	6.04E−08	+ + + +	2.02E−04	+	3.52E−02	+	1.70E−01	+	1.15E−04
cg03500056	*ABAT*	6.19E−08	+ + + +	4.21E−06	+	1.53E−02	+	8.29E−03	+	2.10E−07
cg09182678		6.22E−08	− − − −	1.03E−05	−	1.32E−03	−	5.88E−07	−	1.59E−10
cg02286155		6.39E−08	+ + + +	2.99E−03	+	6.56E−03	+	7.40E−02	+	2.41E−04
cg12593793		7.12E−08	− − − −	1.59E−04	−	8.31E−03	−	2.04E−08	−	1.06E−09
cg23172671		7.47E−08	+ + + +	1.22E−04	+	7.70E−02	+	1.25E−01	+	8.01E−05
cg13139542		7.93E−08	− + + +	1.23E−05	+	1.05E−02	+	3.78E−01	+	2.67E−05
cg02571142	*DKK4*	9.91E−08	+ + + +	6.33E−04	+	4.34E−03	+	6.97E−02	+	5.34E−05
cg21766592	*SLC1A5*	1.07E−07	− − − −	7.69E−03	−	5.41E−01	−	3.33E−04	−	1.34E−04
cg01526748	*FGF12*	1.18E−07	+ + + +	5.32E−04	+	4.17E−04	+	3.32E−02	+	1.11E−05

The full list of the 83 replicated BMI-related differentially methylated CpGs is presented in S3 Table. The four directions of association with BMI for the FHS-LBC cohorts indicate, in order, the two lab batches in the FHS and the LBC1936 and LBC1921 cohorts individually.

ARIC, Atherosclerosis Risk in Communities; BMI, body mass index; Dir, direction of association with body mass index; FHS, Framingham Heart Study; GOLDN, Genetics of Lipid Lowering Drugs and Diet Network; LBC, Lothian Birth Cohort; PIVUS, Prospective Investigation of the Vasculature in Uppsala Seniors.

#### Age and sex interactions among the BMI EWAS findings

Among the 135 discovery CpGs, a significant sex interaction was demonstrated in the discovery cohorts for one unannotated CpG (cg26651978 on Chromosome 17q25.3; <3 kbp from the 3′ end of *LGALS3BP* [*lectin galactoside-binding soluble 3-binding protein*]), and a significant age interaction for one CpG (cg24678869; *DENND4B* [*DENN domain 4B Rab GDP-GTP exchange factor*]) at *p-*value < 3.7 × 10^−4^ (Bonferroni-corrected *p-*value for 135 tests) (S4 Table). The sex interaction identified at cg26651978 (*LGALS3BP*) modestly replicated in the external cohorts (replication meta-analyses *p =* 0.02), with larger regression coefficients and lower *p-*values in stratified models among men than among women (replication meta-analyses *p =* 1.73 × 10^−6^ and 0.002 in men and women, respectively; overall and sex-stratified regression coefficients for each cohort in S5 Table). The age interaction at cg24678869 (*DENND4B*) did not replicate in the external cohorts (replication meta-analyses *p =* 0.9). Due to the narrow age range in PIVUS, however, this interaction was tested only in ARIC and GOLDN (*n =* 3,079).

#### HIF3A locus methylation

Examining a previously identified BMI-related differential methylation at the *HIF3A* locus [28], we demonstrated modest associations with BMI in the FHS-LBC discovery cohorts for the three reported CpGs (*p =* 0.02 for cg22891070, *p =* 0.03 for cg16672562, and *p =* 0.04 for cg27146050; no significant sex interactions). Stratifying models at the median age of 66 y in the FHS (age range too narrow in LBC for stratification) revealed stronger associations in the younger subset and null associations in the older subset (for cg22891070, cg16672562, and cg27146050, *p =* 0.003, *p =* 0.008, and *p =* 0.046, respectively, among participants ≤66 y of age, and *p =* 0.9, *p =* 0.6, and *p =* 0.4, respectively, among participants >66 y of age).

#### Sensitivity models conditioning on cis methylation quantitative trait loci

Sensitivity models conditioning on the top cis-meQTL (selected by lowest *p-*value; ±500 kb from the CpG) in the FHS demonstrated minimal attenuation of the test statistic for the association of BMI, with differential methylation at the majority of CpGs (81/83 [98%]) attenuated by less than 20% (S6 Table).

### Interindividual Variation in BMI and Distribution of Obesity

**Fig 2:**
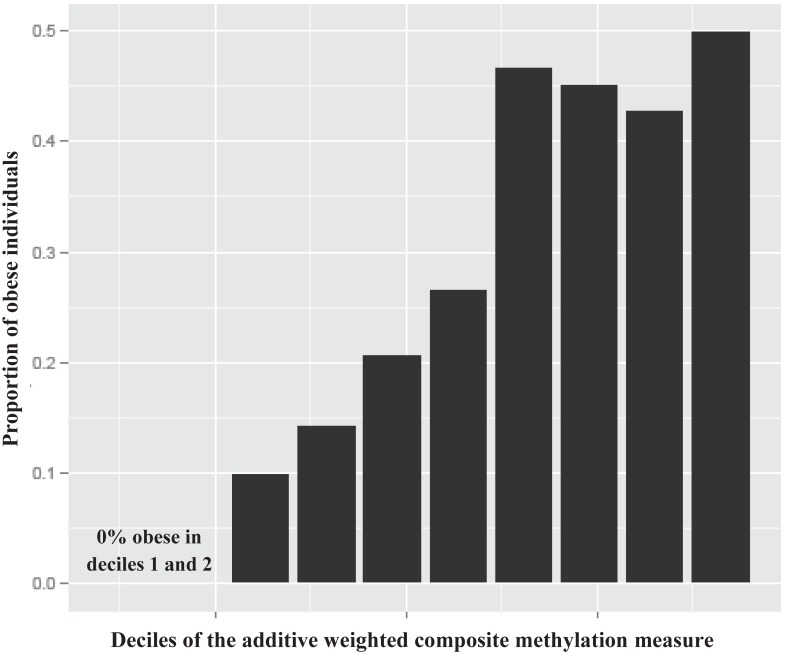
Histogram of the proportion of obese individuals (BMI ≥ 30 kg/m^2^) in the PIVUS cohort across deciles of the additive weighted composite methylation measure of the 77 nonredundant replicated CpGs (|*r*| < 0.7) from the BMI epigenome-wide association study. BMI, body mass index; PIVUS, Prospective Investigation of the Vasculature in Uppsala Seniors.

The interindividual variation in BMI and distribution of obesity captured in the BMI EWAS findings was evaluated. Regressing BMI on the 77 nonredundant (inter-probe correlation |*r*| < 0.7) CpGs from the 83 replicated CpGs identified in the BMI EWAS revealed that 18% of the interindividual variation (adjusted *R*^2^) in BMI is captured by differential methylation beyond age and sex in the external replication cohort PIVUS (S6 Fig). This proportion is similar to that observed when examining a completely independent discovery test set using the 75 CpGs that were methylome-wide significant in the FHS discovery cohort (no replication), which accounted for 17.5% of the interindividual variation in BMI (adjusted *R*^2^) beyond age and sex in the LBCs. Creating an additive weighted composite measure of the 77 nonredundant replicated CpGs and examining the distribution of BMI and obesity (BMI ≥ 30 kg/m^2^) across deciles of the measure demonstrated that the median BMI increased in a graded manner from 22 to 34 kg/m^2^ and the prevalence of obesity rose from 0% to 50% (Figs 2 and S7). For each SD increase in the composite DNA methylation measure in the PIVUS replication cohort, BMI increased by 1.63 (standard error 0.13) kg/m^2^ (*p =* 3.7 × 10^−34^). The odds ratios for obesity (BMI ≥ 30 kg/m^2^) and overweight (BMI 25–29.9 kg/m^2^) compared to the reference group (BMI < 25 kg/m^2^) were 2.8 (95% CI 2.3–3.5; *p =* 1.6 × 10^−25^) and 1.9 (95% CI 1.6–2.2; *p =* 2.5 × 10^−18^), respectively, for each SD increase in methylation measure in age- and sex-adjusted models.

### Three-Way Association of DNA Methylation, Gene Expression, and BMI

We examined the association of DNA methylation at the 83 replicated BMI-related CpGs with gene expression among 2,246 FHS participants, in order to determine which genes in blood may be influenced by differential methylation of the BMI EWAS CpGs. Of the 83 replicated CpGs, annotated gene expression from whole blood was available for 62 CpG–gene expression pairs (three transcript results were unavailable on the microarray, and 18 CpGs were intergenic). There were significant associations (*p-*value < 8 × 10^−4^; 0.05/62) between differential DNA methylation and gene expression in whole blood for 19 CpG–gene expression pairs, representing ten unique gene transcripts (*ABCG1*, *CPT1A*, *SREBF1*, *LGALS3BP*, *DHCR24*, *PHGDH*, *SARS*, *NOD2*, *CACNA2D3*, and *SLC1A5*), with almost all of the CpG–gene expression pairs (18/19; 95%) demonstrating an inverse association of methylation with expression (S7 Table). There were significant three-way associations (CpG versus BMI; CpG versus gene expression; gene expression versus BMI) for 11 CpGs with seven unique annotated genes (Table 4). Five of the seven genes (71%) with significant three-way associations between CpG–gene expression–BMI are known to exhibit cardiometabolic phenotypes in murine gene knockout models [37–44].

**Table 4:** Association results from 11 replicated CpGs with significant three-way associations in whole blood between CpG methylation and BMI, CpG methylation and gene expression, and gene expression and BMI.

Gene (GEx Probe Number)	CpG	CpG versus BMI		CpG versus GEx			GEx versus BMI			Protein Function and Transgenic Mouse Cardiometabolic Phenotype
		Dir	*p*-Value	Dir	*R*^2^	*p*-Value	Dir	*R*^2^	*p*-Value	
*ABCG1* (#3922444)	cg06500161	+	7.6 × 10^−43^	−	0.112	1.2 × 10^−59^	−	0.023	6.2 × 10^−73^	**Function:** membrane transporter for cholesterol and phospholipids; **transgenic mouse:** decreased susceptibility to diet-induced obesity, smaller adipocytes, lower weight [37]
	cg27243685	+	2.3 × 10^−15^	−	0.054	4.4 × 10^−29^	−	0.023	6.2 × 10^−73^	
	cg01881899	+	1.4 × 10^−10^	−	0.035	3.8 × 10^−19^	−	0.023	6.2 × 10^−73^	
	cg10192877	+	1.7 × 10^−08^	−	0.014	1.7 × 10^−08^	−	0.023	6.2 × 10^−73^	
*CACNA2D3* (#2624639)	cg01368219	−	3.2 × 10^−08^	−	0.014	2.9 × 10^−08^	−	0.006	1.1 × 10^−08^	**Function:** voltage-dependent calcium channel complex; **transgenic mouse:** decreased serum free fatty acids [38]
*CPT1A* (#3379644)	cg00574958	−	2.2 × 10^−29^	−	0.025	7.7 × 10^−14^	+	0.017	4.5 × 10^−24^	**Function:** transporter across the mitochondrial inner membrane for fatty acid beta oxidation; **transgenic mouse:** decreased serum glucose and increased serum free fatty acid levels after fasting [39]
	cg17058475	−	2.3 × 10^−09^	−	0.014	2.2 × 10^−08^	+	0.017	4.5 × 10^−24^	
*DHCR24* (#2413907)	cg17901584	−	2.9 × 10^−12^	−	0.017	8.9 × 10^−10^	+	0.003	1.4 × 10^−07^	**Function:** catalyzes the reduction of sterol intermediates during cholesterol biosynthesis; **transgenic mouse:** decreased subcutaneous and mesenteric adipose stores, decreased body size, decreased circulating cholesterol [40]
*SARS* (#2350551)	cg03725309	−	3.9 × 10^−09^	−	0.011	6.6 × 10^−07^	+	0.011	4.7 × 10^−10^	**Function:** catalyzes the transfer of L-serine to tRNA; **transgenic mouse:** no cardiometabolic phenotypes reported
*SLC1A5* (#3866276)	cg02711608	+	6.3 × 10^−08^	−	0.013	5.1 × 10^−08^	+	0.023	1.7 × 10^−69^	**Function:** amino acid transporter; **transgenic mouse:** no cardiometabolic phenotypes reported
*SREBF1* (#3747966)	cg11024682	+	4.8 × 10^−22^	−	0.009	8.0 × 10^−06^	−	0.003	5.2 × 10^−05^	**Function:** transcription factor for sterol biosynthesis; **transgenic mouse:** abnormal fat cell and fat pad morphology, abnormal lipid homeostasis, insulin resistance, enlarged liver [41–44]

Complete list of results for all methylome-wide significant CpGs is available in S8 Table.

BMI, body mass index; Dir, direction of correlation; GEx, gene expression.

### Functional and Regulatory Annotation of the BMI EWAS Findings

#### Gene Ontology pathway analyses

GO analyses of biological process, molecular function, and cellular component pathways of the 55 unique genes annotated to the 83 replicated CpGs (ten CpGs were annotated to genes annotated to other replicated CpGs, and 18 CpGs were intergenic) did not identify any statistically significant pathways after adjustment for multiple testing. Secondarily, in order to further refine gene selection for GO analyses to the genes that demonstrated altered expression, we restricted the GO analyses to the ten unique genes for which variation in expression was associated with differential methylation, as described in the previous section. We identified significant overrepresentation of a biological process pathway in the positive regulation of lipid metabolic processes (GO:0045834; adjusted *p-*value = 0.002; 64-fold enrichment; four overlapping genes [*ABCG1*, *SREBF1*, *CPT1A* and *NOD2*] of 130 total genes in pathway) and two related processes (positive regulation of the cholesterol biosynthetic [GO:0045542] and cholesterol metabolic [GO:0090205] processes; adjusted *p-*value = 0.02–0.03).

#### Regulatory annotation of CpGs associated with gene expression in blood

Most BMI-related CpGs associated with altered gene expression were located within 50 kb of the transcription start site and were within known enhancer or DHSs (S8 and S9 Figs). CpGs associated with BMI were more likely to be in enhancers and DHSs (enrichment *p-*value = 4.5 × 10^−7^ and 9.4 × 10^−4^, respectively) and less likely to reside in CpG islands (depletion *p*-value = 3.2 × 10^−11^) compared to the full set of measured CpGs on the microarray (S8 Table).

#### DNase I hypersensitive site testing of all identified CpGs

Tissue- and cell-type-specific DHS enrichment testing using the eFORGE v1.2 tool demonstrated that the BMI-related CpGs are enriched in DHSs across almost every tissue and cell type assayed in the included ENCODE, BLUEPRINT Epigenome, and Roadmap Epigenomics Project datasets (S10 and S11 Figs), thus supporting the notion that the CpGs identified in blood are also situated in known active regulatory regions in not only blood, but also other metabolically active tissues. Further stratification by whether BMI-related CpGs had overlapping H3 histone methylation revealed that the BMI-related CpGs predominately overlapped regions with mono-methylation and, to a lesser extent, tri-methylation of lysine 4 on histone H3K4 (H3K4me1 and H3K4me3) across numerous tissues from the consolidated Roadmap Epigenomics Project data (S12–S14 Figs). H3K4me1 marks are indicative of enhancers, H3K4me3 marks are indicative of promoters, and both are known markers of transcriptional activation.

### Genetic Instrumental Variable Analyses (Mendelian Randomization)

Successive genetic IV analyses were conducted to infer causal relations between differential methylation, gene expression, and BMI, followed by evaluation of the modeled epigenetic changes on adiposity-related traits using GWAS results (Table 1).

#### Forward Mendelian randomization

Testing the causal association of DNA methylation with BMI revealed that differential methylation at two CpGs had nominally significant causal associations (*p-*value < 0.05) with BMI: (1) cg11024682 (*SREBF1*; cis-meQTL SNP IV rs752579) and (2) cg07730360 (a non-annotated CpG on Chromosome 3q21.3; trans-meQTL SNP IV rs13437553), with causal *p-*value = 0.02 and 0.04, respectively (S15 Fig; S9 Table). Taking forward the two causal CpGs in discovery for external validation, we found that modeled differential methylation at one of the two CpGs (cis-meQTL SNP IV rs752579 for differential methylation at cg11024682 [*SREBF1*]) was associated with BMI in the 2015 GIANT consortium results (*p =* 0.0003; all ancestries).

#### Two-step Mendelian randomization (first step)

In the first step (DNA methylation affecting the mediator, gene expression), the SNP IV (rs752579) utilized in the forward MR analyses to model differential methylation of the *SREBF1* locus (cg11024682) was also found to be strongly associated with altered *SREBF1* gene expression in blood in the FHS (*p =* 3 × 10^−12^; decreased expression in relation to the C allele), a published [45] blood expression quantitative trait locus (eQTL) dataset (*p =* 3.2 × 10^−6^; direction of effect in blood consistent with that seen in the FHS), and liver (*p =* 1 × 10^−15^; in the same direction as observed in blood in a reanalysis of 958 samples [46,47]).

#### Two-step Mendelian randomization (second step)

In the second step (gene expression in blood and alternate tissues affecting BMI), we identified adequate eQTLs for *SREBF1* expression in whole blood (rs1889018; *p =* 1.7 × 10^−15^) from the FHS; in adrenal gland (rs4925138; *p =* 1.1 × 10^−6^) and liver (rs11078366; *p =* 1.8 × 10^−6^) from the Genotype-Tissue Expression (GTEx) Project; and in adipose tissue (rs4985779; *p =* 8.4 × 10^−4^) from the larger MuTHER dataset [48]. The multi-tissue *SREBF1* eQTLs were selected to be largely independent from the *SREBF1* methylation locus SNP IV (details in S1 Methods). We identified significant associations with BMI (adjusted for the four tests, *p* < 0.013) in the GIANT consortium results for two of the four tissue types; specifically, BMI was associated with the SNP IV for *SREBF1* expression in whole blood (rs1889018, *p =* 0.002) and adrenal gland (rs4925138, *p =* 0.0098), but not liver (rs11078366, *p =* 0.89) or adipose tissue (rs4985779, *p =* 0.80).

#### Adiposity-related traits in GWASs

Assessing other cardiometabolic disease associations from published GWASs, the SNP IV (rs752579) for exposure to differential methylation at the *SREBF1* locus (cg11024682) was also found to be associated with (1) adiposity-related traits [49–51] (waist-hip ratio adjusted for BMI [*p =* 2.0 × 10^−4^], adiponectin [*p =* 0.007], birthweight [*p =* 0.046]), (2) diabetes traits [52–55] (type 2 diabetes [*p =* 0.002], fasting insulin adjusted for BMI [*p =* 0.001], HbA1C [*p =* 0.003], HOMA-B [*p =* 0.007]), (3) lipid levels [56] (triglycerides [*p =* 0.001], high-density lipoprotein cholesterol [*p =* 0.03]), and (4) coronary artery disease [57] (*p =* 1.7 × 10^−6^). Additionally, the SNP IV for increased *SREBF1* expression in whole blood (rs1889018) was also associated with waist-hip ratio (*p =* 0.0002), adiponectin (*p =* 0.003), and triglycerides (*p =* 0.02) based on GWAS results [49,50,56]. The SNP IV for increased *SREBF1* expression in the adrenal gland (rs4925138) was also nominally associated with adiponectin (*p =* 0.02), triglycerides (*p =* 0.022), and low-density lipoprotein change in response to statin treatment (*p =* 0.04) [50,56,58].

#### Causal effect estimates

Each SD increase in DNA methylation at the *SREBF1* locus (cg11024682) was predicted to result in a 2.8-kg/m^2^ decrease in BMI in the FHS (modeling the effect of allele C for rs752579). In contrast, the observed relationship between methylation in blood and BMI in the FHS was in the opposite direction: a 1.0-kg/m^2^ increase in BMI per SD increase in DNA methylation at cg11024682. The predicted direction of effect between methylation and BMI is partly derived from the observed direction of effect between the SNP IV and methylation in blood. Previous literature has reported cell-type-dependent QTLs with opposite directions of effect between a SNP and methylation or expression depending on the cell or tissue type examined [59]. As extensive databases of trans-tissue methylation are unavailable, we examined trans-tissue eQTLs for *SREBF1* from the GTEx Portal [60]. A series of eQTLs for *SREBF1* (false discovery rate ≤ 0.05) demonstrate opposite direction of effect between blood versus adrenal gland (*p-*value < 10^−6^) and additional tissues (at *p-*value < 10^−5^) such as skeletal muscle, esophagus, aorta tissue, and tibial nerve (http://www.gtexportal.org/home/bubbleHeatmapPage/SREBF1). Strong eQTLs for *SREBF1* are likely present in adrenal tissue as *SREBF1* is highly expressed in the adrenal gland compared to other tissues (http://www.proteinatlas.org/ENSG00000072310-SREBF1/tissue). For example, rs854764 is a strong eQTL for *SREBF1* in both blood and adrenal tissue but in opposite directions (*p =* 3.8 × 10^−12^ and *p =* 4 × 10^−6^, respectively, in the GTEx catalog) and is associated with BMI in GIANT (*p =* 0.001) and waist-hip ratio (*p =* 9.2 × 10^−4^), adiponectin (*p =* 0.02), HbA1C (*p =* 0.02), type 2 diabetes (*p =* 0.03), triglycerides (*p =* 0.04), and coronary artery disease (*p =* 1.1 × 10^−5^) in GWAS results [4,7,50,52,54,57,61]. This SNP, rs854764, is also a meQTL for *SREBF1* locus methylation at cg11024682 in the FHS (*p =* 2.8 × 10^−18^), but the association with *SREBF1* locus methylation in adrenal gland, the potential tissue of effect, is unknown. See S10 Table for causal effect estimates and confidence intervals for the second step of the two-step MR analyses.

#### Reverse Mendelian randomization

**Fig 3:**
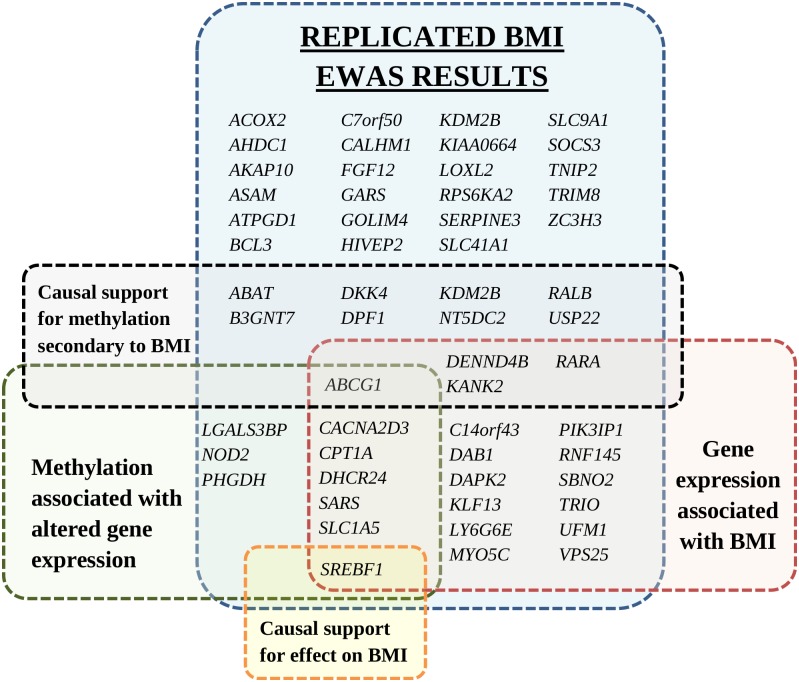
Annotated genes of replicated differentially methylated CpGs identified in the BMI epigenome-wide association study. Genes are grouped by association with gene expression, association of gene expression with BMI, and Mendelian randomization analyses for causal support. Duplicate gene names within the same group are not shown. Figure does not include 18 intergenic CpGs without a gene annotation. BMI, body mass index; EWAS, epigenome-wide association study.

To test whether BMI affects methylation at the identified CpGs, the additive weighted genetic risk score of 97 known BMI SNPs [7] was used as an IV for BMI (*F*-test statistic = 26). Sixteen CpGs were found to be differentially methylated as a consequence of BMI using a nominal causal *p-*value < 0.05 cutoff (full list in S11 Table). The 16 downstream CpGs were annotated to 12 genes (*ABCG1*, *USP22*, *DPF1*, *RARA*, *KDM2B*, *KANK2*, *RALB*, *NT5DC2*, *DENND4B*, *B3GNT7*, *DKK4*, and *ABAT*). A sensitivity analysis using a single SNP in the *FTO* locus as a BMI IV (S12 Table) further supported causal associations downstream of BMI at two of the 16 CpGs (nominal causal *p-*value < 0.05 for cg06500161 and cg04286697, at the *ABCG1* and *B3GNT7* loci, respectively). The annotated genes with BMI-related differential methylation are characterized in Fig 3.

## Discussion

In this analysis of the association of BMI with differential methylation of blood-derived DNA, we provide robust evidence of a connection between replicable epigenetic signaling at 83 CpGs and BMI. We also demonstrate the correlation of BMI-related differential methylation with the altered expression of ten genes in whole blood that are overrepresented in lipid metabolism pathways. Among the 83 replicated BMI-related CpGs, one differentially methylated locus (cg11024682) at the lipid metabolism transcription factor *SREBF1* demonstrated evidence of a causal effect on BMI; genetically predicted exposure to differential methylation and expression of *SREBF1* was found to be associated with BMI and other adiposity traits, glycemic traits, dyslipidemia, and coronary artery disease. In contrast, we found that a substantial proportion (16 out of 83 [19%]) of the BMI-related differentially methylated CpGs identified in this EWAS are likely a consequence of BMI (i.e., downstream signals).

### BMI Variation Is Reflected in DNA Methylation Signatures in Blood

A substantial proportion (~18%) of interindividual variation in BMI is captured by the replicated differentially methylated CpGs in blood. The magnitude of BMI difference (~12 kg/m^2^ between the highest and lowest deciles) equates to substantial health risks; for example, each 5-kg/m^2^ increase in BMI in the general population is associated with a 30% increase in mortality [62].

Our results suggest that epigenetic biomarkers hold the potential to improve risk prediction and help tailor therapy choices to prevent or treat cardiometabolic diseases. For example, at the population level, BMI is an effective measure of average future cardiometabolic disease risk [63], but it is insufficiently predictive at the individual level. Regardless of causality, blood-based biomarkers can be useful for prognostic or diagnostic purposes. Further research is required to determine whether refining BMI-related risk by incorporating epigenetic biomarkers can improve risk prediction and help guide treatment decisions.

### Differential Methylation Is Identified in Loci Known to Be Involved in Adiposity

#### Lipid metabolism

Previously conducted experiments support a causal role of *SREBF1* in adiposity [64]. *SREBF1* (also known as *SREBP1*) plays a central role in energy homeostasis by promoting glycolysis, lipogenesis, and adipogenesis via induction of the conversion of acetyl-CoA to triglycerides (S16 Fig). *SREBF1* promotes the conversion of free fatty acids to triglycerides in the liver and to triglyceride-rich lipoproteins in the bloodstream. In situations of caloric excess, *SREBF1* is a key mediator of the induction of lipogenesis in humans [64]. In mice with diet-induced insulin resistance, inhibition of *SREBF1* attenuates accelerated atherosclerosis, supporting a link to atherosclerosis and coronary artery disease [65]. The causal connection between increased triglyceride-rich lipoproteins and coronary disease is supported by human genetic studies [66]. We highlight the potential role of *SREBF1* expression in the adrenal gland in weight regulation and adiposity-related diseases based on results from the MR analyses. Diseases of the adrenal gland are known to be linked to severe obesity, and adrenalectomy in murine models can reverse genetically induced obesity [67,68]. Our results suggest that altered genomic regulation of *SREBF1* is causally related to BMI; however, the lack of large datasets of meQTLs in numerous tissues and under various conditions, in combination with the inability to conduct tissue-targeted epigenetic editing in relevant experimental models, limits our ability to make a definitive causal inference. Regulation of *SREBF1* is an underexplored target for the prevention of coronary artery disease.

Another of our top genes, *CPT1A* (*carnitine palmitoyltransferase 1A*), is an outer mitochondrial membrane enzyme involved in the utilization of acetyl-CoA, functioning as a key enzyme in the beta-oxidation of long-chain fatty acids in mitochondrial energy metabolism. Acetyl-CoA has recently been identified as a central link between altered lipolysis due to adiposity or inflammation and resultant changes in hepatic insulin resistance with cross-communication between liver and adipose tissue [69]. *ABCG1* (*ATP binding cassette G1*), a cell-membrane lipid transporter, has an established role in reverse cholesterol transport; its role in obesity is supported in previous animal [37] and human studies [70]. *DHCR24* (*24-dehydrocholesterol reductase*) catalyzes the reduction of sterol intermediates during cholesterol synthesis. Differential methylation of *SREBF1*, *CPT1A*, *ABCG1*, and *DHCR24* has been reported in previous EWASs of adiposity, glycemic traits, and lipids [29–31,71–76]. We add to the published literature and provide evidence that differential methylation at the *ABCG1* locus is likely a downstream effect of BMI. From these findings taken together, epigenetic dysregulation is emerging as a common link between obesity and obesity-related comorbidities. Although further functional research is required, we hypothesize that obesity and adiposity-related diseases are partly driven by changes in DNA methylation, with resultant dysregulation of energy balance via effects on expression of lipid metabolism pathway genes. Regulatory mechanisms involved in energy homeostasis have been proposed as attractive targets for the treatment of obesity, metabolic syndrome, and heart disease [77,78]. Our results demonstrate that these connections are evident in humans, adding to previous evidence from animal models [78].

#### Inflammatory pathways

Aside from the lipid metabolism genes, a number of loci involved in inflammatory pathways were identified by our EWAS. Enlarged adipocytes in obese individuals are known to promote inflammation. One BMI-related differentially methylated CpG was identified at the *NOD2* (*nucleotide binding oligomerization domain 2*) locus. *NOD2*, an innate immune receptor, is involved in the immune response to bacterial lipopolysaccharides (LPSs) by activating NF-κB signaling. Uptake of LPS from gut microbiota has been demonstrated to result in increased internalization of LPS-rich lipoproteins into adipocytes and promote macrophage conversion from the M2 form to the inflammatory M1 form [79]. *NOD2* is also included in the GO pathway for regulation of lipid metabolism (0045834) as it is a positive regulator of phosphatidylinositol 3-kinase activity and has been demonstrated to promote vascular inflammation and formation of lipid-rich atherosclerotic lesions in hypercholesterolemic *LDLR*^−/−^ mice [80,81]. *NOD2* interacts with another BMI-related differentially methylated inflammatory gene locus at *SOCS3* (*suppressor of cytokine signaling 3*), a negative regulator of cytokine signaling. In addition, *LGALS3BP* (*lectin*, *galactoside binding soluble 3 binding protein*), also known as *MAC2BP* (*Mac-2-binding protein*), is involved in the immune response associated with lymphokine-activated killer cell cytotoxicity and platelet activation, signaling, and aggregation. *LGALS3BP* has been found to stimulate host defenses and is elevated in individuals with various types of cancer such as breast, lung, colorectal, ovary, and endometrial cancers, many of which are obesity-related. In addition, *LGALS3BP* was recently identified as a promising biomarker for non-alcoholic steatohepatitis and pancreatitis [82,83], known obesity-related diseases. Methylation at the *LGALS3BP* locus demonstrated a significant sex interaction, with a stronger effect in men. This may be related to environmental factors more common in men (such as specific dietary patterns) or male-specific physiology.

### Differential Methylation Is Identified in Loci Not Previously Linked to Adiposity

#### Serine metabolism

Two of the ten genes differentially expressed in association with BMI-related methylation (*PHGDH* and *SARS*) are involved in L-serine metabolism. *PHGDH* (*phosphoglycerate dehydrogenase*) is involved in the early steps of the synthesis of the amino acid L-serine, which plays a role in oxidoreductase as a NADP acceptor in the tricarboxylic acid cycle. *SARS* (*seryl-TRNA synthetase*) catalyzes the transfer of L-serine to tRNA. In addition, *RPS6KA2* (*ribosomal protein S6 kinase A2*), a locus not previously reported as being BMI-related, is a serine/threonine kinase that acts downstream of MAPK signaling and is involved in cell proliferation. L-serine is necessary for specific functions in the central nervous system; however, the link between adiposity and functional health consequences via effects on serine metabolism is currently unknown.

#### Cell-membrane transporters

In addition to the cell-membrane transporters discussed, two additional membrane transporters were identified among the ten genes associated with differential methylation. *SLC1A5* (*solute carrier family 1 member 5*), which was found to have significant three way associations with altered gene expression in blood and BMI, is a sodium-dependent transporter of amino acids. It is activated by insulin concentration, which is often elevated in individuals with obesity. BMI-related differential methylation was also identified at the *CACNA2D3* (*calcium channel*, *voltage-dependent*, *alpha 2/delta subunit 3*) locus. CACNA2D3 is involved in nerve signal transmission and cardiac conduction.

### BMI EWAS Findings in the Context of Published Epigenetic Epidemiology Studies

Previous in silico methods of identifying putative epigenetically regulated obesity genes highlighted *SOCS3* (*suppressor of cytokine signaling 3*) and *RARA* (*retinoic acid receptor alpha*) [84], both of which were identified in the FHS-LBC meta-analysis (*p =* 2.7 × 10^−11^ for cg27637521 in *SOCS3* and *p =* 1.3 × 10^−8^ for cg13274938 in *RARA*). An association study of DNA methylation and BMI in 459 individuals from the Cardiogenics Consortium identified an association of methylation at three CpGs intronic to *HIF3A* (*hypoxia inducible factor 3A*) in blood and adipose cells with BMI [28]. We found modest associations of differential methylation and expression at the *HIF3A* locus with BMI in our study. However, the associations were stronger in younger individuals in the FHS, suggesting that the connection may be less apparent at older ages.

At a nominal causal *p-*value < 0.05, we found that many (16 [19%]) of the replicated CpGs are downstream of BMI. This is consistent with recent findings from longitudinal methylation data and bidirectional MR in the Avon Longitudinal Study of Parents and Children [85] that BMI-related *HIF3A* methylation is likely secondary to differences in BMI.

There is substantial overlap between the identified BMI-related CpGs and reported CpG–metabolite associations in blood from 1,814 participants in the KORA cohort (Kooperative Gesundheitsforschung in der Region Augsburg) [86] (S13 Table). Notably, ceramides and sphingolipids—known to have altered levels among obese individuals and implicated in the development of the metabolic syndrome [87–89]—were identified. In addition, the BMI-related differentially methylated CpG (cg03725309) at the *SARS* locus, as discussed above in the serine metabolism section, was found to be associated with blood levels of serine.

### BMI EWAS Findings in the Context of BMI GWAS Results and Nearby Genetic Variants

Of note, none of the CpGs associated with BMI was near genes previously identified in GWASs of obesity-related traits, such as *FTO* (*fat mass and obesity associated*) or *MC4R* (*melanocortin 4 receptor*). We hypothesize that many of the replicated differentially methylated loci reflect novel pathways involved in the regulation of adiposity or adiposity-related diseases. Long-range interactions of DNA methylation with known obesity-related loci, however, may exist [90]. Further work to understand the role of the novel loci in relation to adiposity is also required. In addition, combining information from DNA methylation with genetic markers identified from DNA sequence variation may allow for improvements in risk prediction previously not possible with sequence variants alone [91].

Many of the significant loci from the discovery phase (73 of 135) were replicated in African-Americans from the ARIC study [30]. Similarly, many of the BMI-related differentially methylated CpGs identified in this study were also reported in relation to BMI in people of Arabic ancestry [34]. In GWASs, failure to replicate across racial/ethnic groups may be due to differences in allele frequencies and linkage disequilibrium patterns. In contrast, the high rate of replication of DNA methylation results for BMI in individuals of European and African and other ancestries suggests that shared environmental exposures or changes secondary to differences in BMI, and not genetic variation, may underlie many of the associations. Further work is needed to identify environmental factors that promote or mitigate disease-relevant obesity-related epigenetic dysregulation. Our analyses that conditioned on top meQTLs showed minimal attenuation, suggesting that the association between differential methylation and BMI is largely independent of genetic variants near the reported CpGs.

### Study Limitations

Our study has several limitations. Results from MR analyses utilizing genetically predicted methylation and expression levels do not prove causation but provide supportive evidence. The results of the MR analyses are based on numerous assumptions, for example, that there are not alternative pathways through which the SNP IV may act on BMI (i.e., pleiotropic effects). The MR assumptions cannot be tested directly and may bias the results. The forward MR results did not reach Bonferroni-adjusted significance thresholds for multiple testing; however, validation of the nominally significant results in the larger GIANT consortium supports our findings. We avoided the use of multi-SNP score IVs as we had already identified adequate single SNP meQTLs and using multi-SNP score IVs would have further risked introducing bias due to pleiotropy. The meQTLs for the MR analyses were derived in the FHS and the outcome was tested within the same cohort, which can potentially result in bias toward significance. The MR analyses, using the blood meQTL IV, suggest an inverse relationship between the predicted methylation of the *SREBF1* locus and BMI, the reverse of the observed relationship, which can be interpreted as a null result. This finding is potentially explained by different directions of effect of QTLs in alternate tissues, which was supported by examining the association of genetic variants in blood versus other metabolically active tissues in the GTEx Project resource. Unfortunately, there are limited datasets of meQTLs in various tissues to explore this further. The observation of associations of BMI with methylation at the same CpG in different directions of effect in blood versus adipose-derived DNA has been previously reported at BMI-related CpG sites [30]. For *SREBF1*, we presume that the metabolic consequences of altered methylation and the effect on BMI occur in tissues other than blood, such as the adrenal gland, with the methylation changes in blood that we were able to detect representing a biomarker of trans-tissue differential methylation [92]. In addition, it is possible that positive and negative feedback loops can result in regulation of the same gene to be both a causal and a downstream effect of adiposity. We would not be able to discern this scenario from the observational cross-sectional data in this study.

An alternate methylation assay would be required for clinical purposes as the current microarrays are unsuitable in a clinical setting. Future research would be required for technical validation for clinical purposes. Our study supports blood cells as a useful accessible tissue for epigenetic biomarker discovery in large population studies. However, our study would not be able to detect tissue-specific methylation changes occurring in non-blood cell lines (e.g., neuron-specific epigenetic modifications in relation to BMI). Many of our top CpGs replicated in the GOLDN study, which assessed DNA methylation in a single blood cell type (CD4+), suggesting that the associations we detected are not likely to be due to confounding by blood cell heterogeneity. Many of the genes associated with BMI-related differential methylation were known to have a role in adiposity and cardiometabolic traits from murine knockout models; however, the universe of knockout models is likely enriched for the study of adiposity and cardiometabolic traits, and we could not directly test whether our results identified more than expected. Our study was conducted among older-age adults, and the findings may not be generalizable to younger ages.

### Conclusions

We provide the results of a large EWAS of BMI in almost 8,000 individuals that identified 83 replicable DNA methylation loci and evidence of complementary transcriptomic differences that were enriched for gene products involved in lipid metabolism. The genetic IV analyses prioritize the *SREBF1* locus for future functional studies to further define the causal relation with adiposity, insulin resistance, obesity-related dyslipidemia, and coronary artery disease. Our findings provide a foundation for further research to determine if individualized epigenetic profiles can be used to guide clinical decision making and improve health outcomes. Our findings may have additional clinical and therapeutic relevance if other loci that are differentially methylated in relation to BMI represent attractive targets for the treatment or prevention of obesity and adiposity-related diseases.

## Supporting Information

S1 Fig Quantile-quantile plot of expected versus observed −log10 *p-*values from the epigenome-wide association study of BMI in the FHS-LBC meta-analysis. Models: (A) age- and sex-adjusted, (B) additionally smoking-adjusted, and (C) additionally excluding frailty/morbid obesity (BMI < 18 kg/m^2^ and > 35 kg/m^2^).
(EPS)

S2 Fig Quantile-quantile plot of expected versus observed −log10 *p-*values from the epigenome-wide association study of BMI in FHS alone. Using surrogate variables to adjust for cell count proportion and technical effects (A) compared to the alternate approach of imputed cell counts and measured technical effects (B). Genomic inflation factor lambda is lower in the surrogate variable analysis approach compared to the approach of imputed cell counts and measured technical effects (1.04 versus 1.25), suggesting fewer potential false positives and a more conservative approach.
(EPS)

S3 Fig Manhattan plot of the epigenome-wide association study of BMI in the FHS-LBC meta-analysis in age- and sex-adjusted models. The dotted line indicates the Bonferroni cutoff for significance of *p-*value < 1.2 × 10^−7^. The top six CpGs with the lowest *p-*values are shown, annotated to their closest gene transcript.
(EPS)

S4 Fig Comparison of −log10 *p-*values of results of the FHS-LBC BMI epigenome-wide association study. (A) Model 1 (age- and sex-adjusted) + Model 2 (additionally smoking-adjusted). (B) Model 1 + Model 3 (excluding BMI < 18 and > 35 kg/m^2^).
(EPS)

S5 Fig Three-dimensional scatterplot of −log10 *p-*values for 135 epigenome-wide significant CpGs from the FHS-LBC discovery cohorts in three external replication cohorts. Replication significance defined as Bonferroni-adjusted *p-*value < 3.7 × 10^−4^ (0.05/135). CpGs significant in one, two, and all three replication cohorts are depicted in green, yellow, and red, respectively. Annotated genes are labeled for CpGs replicated in all three cohorts. Full list of replication results is available in S2 Table.
(EPS)

S6 Fig Variation in BMI explained (adjusted *R*^2^) by differential methylation of 77 nonredundant replicated CpGs in the FHS-LBC epigenome-wide association study and tested in the independent PIVUS cohort. CpGs are added in decreasing order of significance and are adjusted for age, sex, and preceding CpGs.
(EPS)

S7 Fig Boxplot of BMI in the PIVUS cohort across deciles of the additive weighted composite measure of differential DNA methylation at 77 nonredundant replicated CpGs. (EPS)

S8 Fig Relationship between location of CpG relative to the transcription start site and proportion of variation in changes in corresponding gene expression, stratified by whether the CpG resides in a known DHS or enhancer region. CpGs located in known DHS or enhancer regions are depicted in red. bp, base pairs; DHS, DNase I hypersensitive site; TSS, transcription start site.
(EPS)

S9 Fig Relationship between location of CpG relative to the transcription start site and proportion of variation in changes in corresponding gene expression, stratified based on location relative to nearest CpG island. Shores are defined as up to 2 kb from the CpG island, and shelves are defined as up to 2 kb from the CpG shore. TSS, transcription start site.
(EPS)

S10 Fig Enrichment of nonredundant replicated differentially methylated CpGs from the BMI epigenome-wide association study in DNase I hypersensitive sites among various cell and tissue types using ENCODE and 2012 Roadmap Epigenomics Project data. (A) ENCODE and (B) 2012 Roadmap Epigenomics Project.
(EPS)

S11 Fig Enrichment of nonredundant replicated differentially methylated CpGs from the BMI epigenome-wide association study in DNAse I hypersensitive sites among various cell and tissue types using consolidated Roadmap Epigenomics Project and BLUEPRINT Epigenome data. (C) consolidated Roadmap Epigenomics Project and (D) BLUEPRINT Epigenome.
(EPS)

S12 Fig Enrichment of nonredundant replicated differentially methylated CpGs from the BMI epigenome-wide association study in regions overlapping histone modifications in the consolidated Roadmap Epigenomics Project data: H3K4me1 and H3K4me3 histone modifications. Presented is enrichment of BMI EWAS CpGs in regions overlapping (A) H3K4me1 and (B) H3K4me3 histone modifications.
(EPS)

S13 Fig Enrichment of nonredundant replicated differentially methylated CpGs from the BMI epigenome-wide association study in regions overlapping histone modifications in the consolidated Roadmap Epigenomics Project data: H3K9me3 and H3K27me3 histone modifications. Presented is enrichment of BMI EWAS CpGs in regions overlapping (C) H3K9me3 and (D) H3K27me3 histone modifications.
(EPS)

S14 Fig Enrichment of nonredundant replicated differentially methylated CpGs from the BMI epigenome-wide association study in regions overlapping histone modifications in the consolidated Roadmap Epigenomics Project data: H3K36me3 histone modifications. Presented is enrichment of BMI EWAS CpGs in regions overlapping (E) H3K36me3 histone modifications.
(EPS)

S15 Fig Depiction of an example result for SREBF1 from the bidirectional Mendelian randomization analyses for each of the replicated CpGs and BMI. Example shown illustrates the bidirectional relationship of cg11024682 intronic to SREBF1 and BMI using a meQTL to model the exposure of differential methylation at that locus and an additive weighted genetic risk score using known BMI-related SNPs to model the exposure of elevated BMI.
(EPS)

S16 Fig DNA methylation and mRNA expression of CPT1A and SREBF1 in whole blood in triglyceride and fatty acid catabolism (beta-oxidation) pathways was observed in association with higher BMI. (EPS)

S1 Methods Supplemental methods. (DOCX)

S1 STROBE Checklist (DOC)

S1 Table Complete list of methylome-wide significant (*p-*value < 1.2 × 10^−7^) CpGs associated with BMI in the FHS-LBC meta-analysis. (XLSX)

S2 Table Secondary models including additional adjustment for smoking and exclusion of BMI < 18 and > 35 kg/m^2^. (XLSX)

S3 Table External replication of methylome-wide significant CpGs. (XLSX)

S4 Table Distribution and variability of replicated BMI-related differentially methylated CpGs. (XLSX)

S5 Table Secondary models testing age and sex interactions. (XLSX)

S6 Table Sex-stratified models for cg26651978 (*LGALS3BP*) in the replication cohorts. (XLSX)

S7 Table Association of BMI with the replicated CpGs conditional on the top methylation QTL in the FHS. (XLSX)

S8 Table Three-way association results of CpGs, expression levels of nearby annotated genes, and BMI. (XLSX)

S9 Table Enrichment of BMI-related CpGs associated with gene expression in DNase I hypersensitive sites and enhancers. (XLSX)

S10 Table Results from the forward Mendelian randomization (DNA methylation affecting BMI) for the 83 replicated CpGs. (XLSX)

S11 Table Results from the mediator-to-outcome analyses of the two-step trans-tissue Mendelian randomization. (XLSX)

S12 Table Results from the reverse Mendelian randomization for the 83 replicated CpGs using the BMI genetic risk score instrumental variable. (XLSX)

S13 Table Sensitivity analyses for the reverse Mendelian randomization for the 16 implicated CpGs using the *FTO* locus SNP instrumental variable. (XLSX)

S14 Table Overlap between replicated BMI-related CpGs and metabolites as reported from the KORA cohort [86]. (XLSX)

S1 Text Project proposal. (DOCX)

## Abbreviations

ARIC: Atherosclerosis Risk in Communities
BMI: body mass index
DHS: DNase I hypersensitive site
eQTL: expression quantitative trait locus
EWAS: epigenome-wide association study
FHS: Framingham Heart Study
GIANT: Genetic Investigation of ANthropometric Traits
GO: Gene Ontology
GOLDN: Genetics of Lipid Lowering Drugs and Diet Network
GTEx: Genotype-Tissue Expression
GWAS: genome-wide association study
IV: instrumental variable
LBC: Lothian Birth Cohort
meQTL: methylation quantitative trait locus
MR: Mendelian randomization
PIVUS: Prospective Investigation of the Vasculature in Uppsala Seniors
QTL: quantitative trait locus
SD: standard deviation
SNP: single nucleotide polymorphism
