# Supplementary material for: Association of Body Mass Index with DNA Methylation and Gene Expression in Blood Cells and Relations to Cardiometabolic Disease: A Mendelian Randomization Approach
Source: PLoS Med. 2017 Jan 17;14(1):e1002215. doi: 10.1371/journal.pmed.1002215 (PMC5240936; doi:10.1371/journal.pmed.1002215)
Supplement: S1 Methods — (DOCX) [file pmed.1002215.s017.docx]

**SUPPLEMENTAL METHODS**

**Anthropometric and covariate measurements**

In FHS, weight was measured to the nearest pound with the participant wearing only a gown without slippers or shoes, standing in the middle of the scale (Detecto Scale, Worchester Scale) with weight equally distributed on both feet. Standing height was measured to the nearest ¼ inch, with the participant barefoot or wearing thin socks, using a vertical mounted stadiometer. Weight and height were measured in both LBC studies using a standardized protocol by a trained nurse. Participants were asked to remove their shoes before a SECA stadiometer was used to assess height in centimeters. Weight (after removing shoes and outer clothing) was measured in kilograms using a digital readout from electronic SECA scales.

Self-reported cigarette smoking was categorized based on current smoking status (current smoker, non-current smoker) in the FHS and into three categories (current smoker, never smoker, and former smoker) in the LBC.

**DNA methylation assays**

*Framingham Heart Study Offspring Cohort*: In FHS, buffy coat preparations were obtained from the whole blood samples. Genomic DNA was extracted using the Gentra Puregene DNA extraction kit (Qiagen, Venlo, Netherlands) which subsequently underwent bisulfite conversion using the EZ DNA Methylation kit (Zymo Research, Irvine, CA). Samples underwent whole genome amplification, fragmentation, array hybridization, and single-base pair extension. DNA methylation arrays were run in two laboratory batches at the Johns Hopkins Center for Inherited Disease Research (lab batch #1) and University of Minnesota Biomedical Genomics Center (lab batch #2). The first batch included 576 samples from an earlier cardiovascular disease (CVD) case-control study[^1^](#_ENREF_1) and the second batch included 2270 samples from the remainder of the Offspring cohort participants. DNA methylation results underwent normalization within laboratory batches using the DASEN methodology implemented in the *wateRmelon* package[^2^](#_ENREF_2) in R (version 3.0.2), which includes background adjustment of the methylated and unmethylated intensities and quantile normalization of the methylated and unmethylated probes within the two types of probe technologies separately. For sample quality control, we excluded samples with a missing rate >1% at p<0.01 (n=10 for batch #1 and n=35 for batch #2), poor single nucleotide polymorphism (SNP) matching to the 65 SNP control probe locations (n=38 for batch #1 and n=41 for batch #2), and outliers by multi-dimensional scaling techniques (n=25 for batch #1 and n=48 for batch #2). For quality control at the probe level, we excluded probes with a missing rate >20% at p<0.01 (n=466 from batch #1 and n=366 from batch #2), as well as probes previously identified to map to multiple locations[^3^](#_ENREF_3) or to have an underlying SNP (minor allele frequency >5% in European ancestry (EUR) 1000 genomes project data) at the CpG site or within 10 bp of the single base extension (n=42,251). This left 2377 FHS participants with phenotype data and 443,252 probes available for analyses. The FHS methylation data are available at dbGaP under the accession number phs000724.v2.p9.

*Lothian Birth Cohorts of 1936 and 1921*: In LBC, extraction of DNA from whole blood was carried out on 514 LBC1921 and 1,004 LBC1936 samples at the MRC Technology, Western General Hospital, Edinburgh (LBC1921) and the Wellcome Trust Clinical Research Facility (WTCRF), Western General Hospital, Edinburgh (LBC1936). Typing of 485,512 CpG sites was carried out at the WTCRF where bisulfite converted DNA samples (Zymo Research, Irvine, CA) were hybridized to the 12 sample Illumina HumanMethylation450BeadChips[^4^](#_ENREF_4) using the Infinium HD Methylation protocol and Tecan robotics. Background correction of the raw intensity data and generation of the methylation beta values was done using the R *minfi* package.[^5^](#_ENREF_5) Quality control steps included the removal of probes with a low (<95%) detection rate at p<0.01. Array control probes were inspected manually and low quality samples (e.g. samples with inadequate hybridization, bisulfite conversion, nucleotide extension or staining signal) were removed. Samples with a low call rate according to the Illumina-based threshold (samples with <450,000 probes detected at p<0.01) were removed. Both LBC studies have been genotyped using the Illumina 610-Quadv1 genotyping platform so genotypic information from the 65 SNP control probes on the methylation chip were cross-validated with those from the genotyping chip using the *wateRmelon* package.[^2^](#_ENREF_2) Where there was low correspondence, samples were excluded (n=9). We also excluded 8 participants whose reported sex did not match their predicted sex, based on XY probes. Finally, a generalized linear model with a logistic link function was used to adjust the beta value of each probe for technical variation (plate, array, position on the chip, and hybridization date). The residuals from these models were carried forward as the dependent variables of interest. LBC methylation data have been submitted to the European Genome-phenome Archive under accession number EGAS00001000910; phenotypic data are available at dbGaP under the accession number phs000821.v1.p1.

**Gene expression assays**

In the FHS, RNA was extracted from whole blood using the PAXgene Blood RNA System Kit (Qiagen, Venlo, Netherlands) with mRNA expression profiling from the Affymetrix Human Exon 1.0 ST GeneChip platform. Robust multichip average (RMA) methods[^6^](#_ENREF_6) were used to normalize the gene expression values with quality control measures as previously reported.[^1^](#_ENREF_1) Cell count proportions were derived from gene expression markers in this sample set as there was overlap between gene expression measures and directly measured cell counts (lymphocytes, monocytes, neutrophils, basophils, and eosinophils) from a sample of 2,280 Third Generation FHS participants obtained during the second examination cycle (2008-2011). Internal validation using training and testing datasets achieved an r^2^ > 0.8 in the majority of cell lines (except basophils). Imputed cell counts were used in the gene expression analyses but not in the DNA methylation analyses (which used surrogate variable analyses to adjust for cell count) as there were 131 samples with DNA methylation but no gene expression.

**Methylome-Wide Association Study (methWAS) methods**

In the FHS, inverse-normal transformation of the CpG probe beta value (the ratio of methylated probe intensity and total probe intensities) was specified as the dependent variable. The CpG beta value was transformed to reduce the influence of outliers. BMI was specified as the independent variable with adjustment for age and sex as fixed effects and family structure as a random effect. Families were clustered based on shared pedigrees, which included unrelated spouses, as a conservative approach to additionally account for shared environmental (i.e. household) factors. In the FHS, surrogate variable analysis[^7^](#_ENREF_7)^,^[^8^](#_ENREF_8) (SVA) was used to adjust for unmeasured technical and batch effects, including cell count composition. SVA is a well-established method to account for unmeasured technical effects in high-dimensional data and has been demonstrated to account for differential cell composition in DNA methylation data.[^9^](#_ENREF_9) Using SVA to adjust for cell count proportion and technical effects were compared to adjusting for imputed cell count using the Houseman method[^10^](#_ENREF_10) and measured technical covariates for chip, row, a column on the array as random effects in the linear mixed effects regression model. Adjusting for SVAs resulted in a lower genomic inflation factor lambda (1.04 vs. 1.25 in FHS alone) compared to imputed cell counts and measured technical effects in the primary model. There was a correlation of r=0.86 between test-statistics in the two approaches with top results remaining similar (not presented). Using SVA to adjust for cell count proportion and technical effects was deemed the more conservative approach and therefore utilized. Complete blood cell counts were measured in LBC[^11^](#_ENREF_11). In FHS, regression models were run within laboratory batches separately. Robustness of primary models were examined by additionally adjusting for CVD in FHS only (due to the case-control nature of laboratory batch #1), which resulted in little change to the results (not presented). Comparison of the primary model run with and without adjusting for family structure in the FHS shows very high correlation of resulting beta-coefficient (r=0.97).

Secondary models additionally adjusted for smoking status, and excluded those with BMI <18kg/m^2^ and >35kg/m^2^ (in order to reduce confounding due to frailty or morbid obesity and obesity-related diseases). BMI was used as the dependent variable in sensitivity analyses (not presented) with negligible differences in results when BMI was specified as the dependent versus independent variable. For the methylome-wide significant CpGs from the FHS and LBC meta-analysis, age*BMI and sex*BMI interaction terms were examined separately in the age-, sex-, and smoking-adjusted models.

**External replication cohorts**

*Atherosclerosis Risk in Communities (ARIC):* The ARIC Study is a prospective cohort

study of cardiovascular disease risk in four U.S. communities.[^12^](#_ENREF_12) Between 1987 and 1989,

7,082 men and 8,710 women aged 45–64 years were recruited from Forsyth County, North

Carolina; Jackson, Mississippi (African Americans only); suburban Minneapolis, Minnesota; and

Washington County, Maryland. The ARIC Study protocol was approved by the institutional review board of each participating university. After written informed consent was obtained, including that for genetic studies, participants underwent a baseline clinical examination (Visit 1) and four subsequent follow-up clinical exams (Visits 2 – 5). Methylation assays were performed on African Americans only, and the present study comprises a cross-sectional analysis of these data. DNA samples for the methylation analysis presented here had been collected at Visit 2.

Measurements of BMI and other covariates also come from Visit 2. Anthropometric measurements were taken with the subject wearing a scrub suit and no shoes. White blood cell (WBC) count was assessed by automated particle counters within 24 hours after venipuncture in local hospital hematology laboratory. The reliability coefficient for the WBC count measurement was greater than 0.96. Genomic DNA was extracted from peripheral blood leukocyte samples using the Gentra Puregene Blood Kit (Qiagen; Valencia, CA, USA) according to the manufacturer’s instructions (www.qiagen.com). Bisulphite conversion of 1 ug genomic DNA was performed using the EZ-96 DNA Methylation Kit (Deep Well Format) (Zymo Research; Irvine, CA, USA) according to the manufacturer's instructions ([www.zymoresearch.com](http://www.zymoresearch.com)). Bisulfite-converted DNA was used for hybridization on the HumanMethylation450 Bead Chip, following the Illumina Infinium HD Methylation protocol ([www.illumina.com](http://www.illumina.com)). The analysis of the ARIC data used Beta MIxture Quantile dilation (BMIQ)[^13^](#_ENREF_13) to normalize type 2 design probes to approximate the statistical distribution of type 1 probes. Positive and negative controls and sample replicates were included on each 96-well plate assayed. After exclusion of controls, replicates, and samples with integrity issues or that failed bisulfite conversion, a total of 2,841 study participants had HM450 data available for further quality control analyses. We removed poor-quality samples with pass rate <99%, that is, if the sample had at least 1% of CpG sites with detection p-value > 0.01 or missing (N=37), indicative of lower DNA quality or incomplete bisulfite conversion, and samples with a possible gender mismatch based on evaluation of selected CpG sites on the Y chromosome (N=2), leaving a total of 2,802 samples available for analysis. At the target level, we flagged poor-quality CpG sites with average detection p-value > 0.01, and calculated the percentage of samples having detection p-value > 0.01 for each autosomal and X chromosome CpG site. Of the 2,802 samples with DNA methylation data after quality control, 50 were missing both concurrent body mass index and waist circumference. Removal of subjects without complete covariate data needed for confounder adjustment (predominantly white blood cell count) yielded a final data set of n=2,096 individuals for the BMI analysis. For replication in ARIC, cross-sectional linear mixed models were tested with methylation β-values as the dependent variable, the chip (array) specified as a random effect and the following variables specified as fixed effects: standardized adiposity variable (BMI with mean= 0 and standard deviation (SD)=1), sex, age, study center, WBC count, education, household income, current cigarette smoking, current alcohol consumption, leisure time physical activity, leukocyte cell type proportions (neutrophils, lymphocytes, monocytes, eosinophils) imputed using the algorithm developed by Houseman and colleagues,[^14^](#_ENREF_14) and 10 principal components scores (PCs) from the Illumina Infinium HumanExome Beadchip genotype array, to account for potential confounding by genetic ancestry.[^15^](#_ENREF_15)

*Genetics of Lipid Lowering Drugs and Diet Network (GOLDN):* The Genetics of Lipid Lowering Drugs and Diet Network (GOLDN) study, described in detail in prior publications,[^16^](#_ENREF_16) recruited families with at least two siblings from the participants of the National Heart, Lung, and Blood Institute Family Heart Study at the Minneapolis and Salt Lake City sites. The study protocol was approved by the Institutional Review Boards at the University of Minnesota, University of Utah, and Tufts University/New England Medical Center, and written informed consent was obtained from all participants. CD4+ T-cells were isolated from frozen buffy coat samples isolated from peripheral blood collected at the baseline visit (prior to intervention). DNA was extracted using DNeasy kits (Qiagen, Venlo, Netherlands). 500ng of each DNA sample was treated with sodium bisulfite (Zymo Research, Irvine, CA) and methylation was measured using the Infinium Human Methylation 450 array (Illumina, San Diego, CA) as previously described. Probes with an associated detection p-value >0.01 and samples with more than 1.5% missing data points across ~470,000 autosomal CpGs were excluded. CpG probes that failed QC (p-value >0.01) in >10% of samples and CpGs where the probe sequence mapped either to a location that did not match the annotation file or to more than one locus were likewise excluded from analysis.[^17^](#_ENREF_17) The filtered β scores were normalized using the ComBat package for R software.[^18^](#_ENREF_18) Normalization was performed on random subsets of 10,000 CpGs per run, with each array of 12 samples used as a "batch." Probes from the Infinium I and II chemistries were separately normalized and β scores for Infinium II probes were then adjusted using the equation derived from fitting a second order polynomial to the observed methylation values across all pairs of probes located <50bp apart (within-chemistry correlations >0.99), where one probe was Infinium I and one was Infinium II. Associations between methylation scores at each CpG site and BMI were tested using linear mixed models, adjusted for age, sex, study site, the first 4 principal components generated to capture T-cell purity as fixed effects, and pedigree as a random effect using the *lmekin* function of the kinship package in R.

*Prospective Study (PIVUS):* The PIVUS study is a prospective community-based cohort of participants from Uppsala, Sweden.[^19^](#_ENREF_19) Baseline medical examination (including anthropometric measurements) was collected at 70 years of age (2001-2004). Blood for DNA methylation assays were collected at the baseline visit. Genomic DNA was extracted from whole blood samples and bisulphite conversion of 500 ng genomic DNA was performed using the EZ-96 DNA Methylation Gold Kit from Zymo Research Product. The equivalent of approximately 200ng of bisulphite-converted DNA was removed, evaporated to a volume of <4μl, and used for methylation profiling using the Illumina Infinium assay and the Illumina HumanMethylation450_v.1.2 bead chip according to the protocol from the supplier. The results were analyzed with GenomeStudio 2011.1 from Illumina Inc. After exclusion of replicates, a total of 1002 study participants had methylation data available for quality control procedures. Three samples were excluded based on poor bisulphite conversion efficiency, twelve samples due to low pass rate of CpG sites (<98.5% with a detection p-value >0.01) and a further six samples based on low SNP genotype match (>1 SNP mismatches) between genotypes from the methylation array and Omni/Metabochip genotyping chips leaving 981 samples. Following removal of participants with abnormal leukocyte cell counts (>10x10^9^ cells/L; n=14) a final set of 967 individuals were used in BMI analysis. The signal intensities for the methylated and unmethylated states were then quantile normalized for each probe type separately, and beta values were calculated. Association between methylation beta values at each CpG site and BMI were analyzed using linear models adjusted for sex, age, predicted white cell counts (Houseman algorithm), bisulphite conversion plate (96-well plate) and bisulphite conversion efficiency mean (calculated from control probes).

**Identification of cis-methylation Quantitative Trait Loci (meQTL)**

The association between DNA methylation and single nucleotide polymorphisms (SNPs) within +/-500 kb from the replicated CpGs identified in the BMI methWAS were tested in linear models in the FHS. The residuals of DNA methylation after adjustment for the fixed (age, sex) and random effects (technical covariates), were taken forward and specified as the dependent variable. Allele dosage of all 1000G imputed SNPs (imputation Rsq > 0.8 and MAF > 0.1%) residing within 500kb of the CpG were individually tested and specified as the dependent variables of interest in linear models adjusting for the kinship correlation structure. The single top meQTL for the conditional sensitivity models were selected by choosing the meQTL SNP with the lowest p-value for the association with DNA methylation at the CpG of interest.

**Gene expression analyses**

The association between the DNA methylation and gene expression (available in 2246 FHS participants with DNA methylation) was performed on the residuals after the removal of the fixed and random covariates, along with the kinship correlation structure using a linear model, primarily to avoid potential confounding by blood count. Only CpGs that were methylome-wide significant were tested and individual CpGs were tested against each single annotated transcript in the regression model. We used the gene expression transcript annotated to that CpG in the Illumina manifest which is based on tight proximity of the CpG to the coding region. As trans-networks of transcriptome-wide gene expression changes associated differential methylation have not yet been well-defined and replicated, only differential CpG methylation relations to single annotated gene expression were considered in these analyses.

Cell count proportions were derived in the FHS using data from the 2,2285 participants in the FHS Third Generation cohort with both gene expression and whole blood complete blood cell counts (CBCs, Beckman Coulter, Brea, California). The cell counts of the remaining samples were imputed using a Partial Least Square (PLS) method using 2/3 (n=1,523) of the gene expression data as the training set. The prediction and cross-validated estimates are computed on the remaining 1/3 of the data. Cross-validated estimates of prediction accuracy (R^2^) for the CBC components (WBC, RBC, platelet, neutrophil percent, lymphocyte percent, monocyte percent, eosinophil percent and basophil percent) were 0.61, 0.41, 0.25, 0.83, 0.83, 0.81, 0.89, and 0.25, respectively. We conducted comparisons between results of using imputed cell counts and those of using measured ones and did not find significant difference. Thus, we used measured cell counts when available and imputed ones when not.

**Bidirectional and two-step Mendelian randomization (MR) of methWAS findings**

Evidence for a causal contribution of differential methylation contributing to BMI (forward direction) and elevated BMI affecting DNA methylation (reverse direction) was assessed using a two-stage least squares (2SLS) approach [^20^](#_ENREF_20) where SNPs were used to derive genetic sequence driven changes in DNA methylation (for the forward direction analysis), or BMI (for the reverse direction analysis), for participants from one of the discovery cohorts (FHS).

Forward direction MR (DNA methylation contributing to elevated BMI): The single top methylation quantitative trait locus (meQTL) determined by lowest p-value for each CpG derived from whole blood in the FHS was used as an instrumental variable (IV) for lifelong exposure to differential methylation in blood at each of the replicated MWAS CpGs. We used a single SNP IV instead of a multi-SNP risk score in order to reduce bias from potential unknown pleiotropic effects from some of the included SNPs. SNP IVs for differential methylation were comprised of the single top methylation quantitative trait loci (meQTLs) based on the lowest p-value for each of the replicated methWAS CpGs. mQTLs were derived in the FHS using linear mixed effect regression models of genome-wide SNPs (1000G imputation; imputation Rsq > 0.8) on the replicated CpGs adjusted for age, sex, SVAs and family structure. For the single top mQTL for each of the replicated CpGs, we quantified the first-stage F-statistic to inform the strength of the genetic instrument to ensure an F-test > 10 (to reduce bias due to weak instruments). For CpGs causally implicated in influencing BMI using a trans-mQTL (> 500 kb from the CpG or different chromosome) IV, for which pleitropic effects may drive the effect of the SNP on differential methylation and risk violating one of the MR assumptions, a second analysis selecting the top cis-mQTL (within +/- 500 kb of the CpG) was attempted to support the initial results. As compared to the cross-sectional association analyses, the MR analyses are less prone to reverse causation and confounding.

The first stage of the 2SLS involves using a linear regression of the DNA methylation on the meQTL SNP IVs and saving the predicted values of DNA methylation for each participant expected based on their mQTL genotype (ie. the lifelong site-specific DNA methylation exposure value for that participant due to genetic sequence variation). In the second stage, BMI is then regressed on the predicted value of site-specific DNA methylation. The regression coefficient obtained in the second stage can be interpreted as being the causal effect of site-specific differential DNA methylation on BMI. The MR analyses were conducted using the *ivreg* function in STATA adapted to run in R.

In the second stage of the 2SLS, causal effect estimates and p-values were derived from the association of the genetically predicted site-specific differential methylation and BMI, incorporating the error from the first stage. We first conducted the MR analysis in one of the discovery cohorts (FHS) and considered causal p-values meeting Bonferroni cutoff for the number of CpGs tested as significant. The MR analyses were limited in power for SNP associations in the sample sizes assembled for this study. Therefore if no genetically predicted CpG methylation values were causally related to BMI at strict Bonferonni corrected p-value cutoffs, we took forward predicted CpG methylation implicated to influence BMI with a nominal causal p-value <0.05 for external validation in the 2015 GIANT (Genetic Investigation of ANthropometric Traits) consortium genome-wide association study (GWAS) results from meta-analysis of 339,224 individuals [^21^](#_ENREF_21).

Two step MR (DNA methylation influencing gene expression affecting BMI): The two step MR attempts to infer whether the hypothesized mediator (gene expression) is causally influenced by the exposure (DNA methylation) and, second, whether the mediator (gene expression) causally affects the outcome (BMI). This allows us to infer whether the annotated gene to the BMI-related differentially methylated CpG is the mediator of effect, and secondarily, to examine differential expression in multiple tissues as the mediator of effect on BMI. As external coefficients were utilized in this analysis, a 2SLS approach could not be used and instead causal effect estimates were determined by the ratio of coefficients (Wald ratio) method and confidence intervals by the Fieller’s theorem which includes a normality assumption [^20^](#_ENREF_20)^,^[^22^](#_ENREF_22).

In the first step, SNP IVs for differential methylation (the single top mQTL for each replicated MWAS CpG with support for a causal effect on BMI in the forward MR analyses) were assessed for associations with expression of the annotated gene in order to determine if the SNP was also an expression QTL (eQTL) in blood (in the FHS and external blood eQTL dataset [^23^](#_ENREF_23)), adipose and liver (from published literature [^24-26^](#_ENREF_24)) in addition to being an mQTL.

In the second step, the top eQTL (by lowest p-value) independent from the mQTL (SNPs with linkage disequilibrium r^2^ < 0.3 in HapMap CEU populations or > 500kb apart) were obtained for the genes of interest (selected based on the annotated genes of the CpGs implicated as causally affecting BMI from the forward MR analyses). In this study, eQTLs from metabolically active tissues were obtained from published eQTL datasets and the GTeX (Genotype-Tissue Expression) project resource [^26^](#_ENREF_26). The independent eQTLs were tested in association with BMI and cardiometabolic phenotypes using results from the GIANT consortium and GRASP GWAS catalog in order to examine the mediators’ causal effect of expression of the genes of interest in metabolically active tissues.

Extension of MR findings to multiple tissues and adiposity-related traits: In order to support relevance in additional biologically active tissues, the SNP IV to methylation associations from the GIANT-validated BMI-related causal CpGs were assessed using external mQTL data from the published literature (liver tissue in 181 samples [^24^](#_ENREF_24)) and GENEVAR (GENe Expression Variation [^25^](#_ENREF_25)) to examine mQTLs in adipose tissue from 856 healthy female twins of the MuTHER (Multiple Tissue Human Expression Resource) resource [^27^](#_ENREF_27). Second, in order to support the relevance of the MR findings to additional cardiometabolic disease phenotypes, the significant SNP IVs implicating a causal relationship between site-specific differential methylation and BMI were interrogated in the GRASP (Genome-Wide Repository of Associations Between SNPs and Phenotypes) catalog of published GWAS study results [^28^](#_ENREF_28).

Reverse direction MR (elevated BMI contributing to differential methylation): We used the 97 genome-wide significant SNPs associated with BMI from the 2015 GIANT consortium results as an additive weighted genetic risk score as the IV for BMI in a 2SLS approach, as described in the previous section, to identify loci with methylation changes that occur secondary to BMI differences. If no results met Bonferonni correction for multiple testing, we considered nominal causal p-values <0.05 as significant. In the reverse direction, SNP IVs for BMI were obtained from the 2015 GIANT consortium results utilizing the 97 SNPs with genome-wide significant associations with BMI in all ancestries. The 2SLS approach as described above was utilized using the BMI SNPs to impute a genetically predicted BMI for each individual and then each of the replicated CpGs is regressed on the predicted BMI. As a sensitivity analyses, a SNP in the FTO locus was used as an instrument for BMI which is less prone to pleiotropic effects than a BMI genetic risk score but also less powerful to detect causal associations.

**References for Supplemental Methods**

1. Joehanes, R.*, et al.* Gene expression signatures of coronary heart disease. *Arterioscler Thromb Vasc Biol* **33**, 1418-1426 (2013).

2. Pidsley, R.*, et al.* A data-driven approach to preprocessing Illumina 450K methylation array data. *BMC Genomics* **14**, 293 (2013).

3. Chen, Y.A.*, et al.* Discovery of cross-reactive probes and polymorphic CpGs in the Illumina Infinium HumanMethylation450 microarray. *Epigenetics* **8**, 203-209 (2013).

4. Bibikova, M.*, et al.* High density DNA methylation array with single CpG site resolution. *Genomics* **98**, 288-295 (2011).

5. Aryee, M.J.*, et al.* Minfi: a flexible and comprehensive Bioconductor package for the analysis of Infinium DNA methylation microarrays. *Bioinformatics* (2014).

6. Irizarry, R.A.*, et al.* Exploration, normalization, and summaries of high density oligonucleotide array probe level data. *Biostatistics* **4**, 249-264 (2003).

7. Leek, J.T. & Storey, J.D. Capturing heterogeneity in gene expression studies by surrogate variable analysis. *PLoS Genet* **3**, 1724-1735 (2007).

8. Leek, J.T., Johnson, W.E., Parker, H.S., Jaffe, A.E. & Storey, J.D. The sva package for removing batch effects and other unwanted variation in high-throughput experiments. *Bioinformatics* **28**, 882-883 (2012).

9. Liang, L. & Cookson, W.O. Grasping nettles: cellular heterogeneity and other confounders in epigenome-wide association studies. *Hum Mol Genet* (2014).

10. Houseman, E.A., Kelsey, K.T., Wiencke, J.K. & Marsit, C.J. Cell-composition effects in the analysis of DNA methylation array data: a mathematical perspective. *BMC Bioinformatics* **16**, 95 (2015).

11. McIlhagger, R.*, et al.* Differences in the haematological profile of healthy 70 year old men and women: normal ranges with confirmatory factor analysis. *BMC Blood Disord* **10**, 4 (2010).

12. The Atherosclerosis Risk in Communities (ARIC) Study: design and objectives. The ARIC investigators. *Am J Epidemiol* **129**, 687-702 (1989).

13. Teschendorff, A.E.*, et al.* A beta-mixture quantile normalization method for correcting probe design bias in Illumina Infinium 450 k DNA methylation data. *Bioinformatics* **29**, 189-196 (2013).

14. Houseman, E.A.*, et al.* DNA methylation arrays as surrogate measures of cell mixture distribution. *BMC Bioinformatics* **13**, 86 (2012).

15. Grove, M.L.*, et al.* Best practices and joint calling of the HumanExome BeadChip: the CHARGE Consortium. *PLoS One* **8**, e68095 (2013).

16. Lai, C.Q.*, et al.* Fenofibrate effect on triglyceride and postprandial response of apolipoprotein A5 variants: the GOLDN study. *Arterioscler Thromb Vasc Biol* **27**, 1417-1425 (2007).

17. Absher, D.M.*, et al.* Genome-wide DNA methylation analysis of systemic lupus erythematosus reveals persistent hypomethylation of interferon genes and compositional changes to CD4+ T-cell populations. *PLoS Genet* **9**, e1003678 (2013).

18. Johnson, W.E., Li, C. & Rabinovic, A. Adjusting batch effects in microarray expression data using empirical Bayes methods. *Biostatistics* **8**, 118-127 (2007).

19. Lind, L., Fors, N., Hall, J., Marttala, K. & Stenborg, A. A comparison of three different methods to evaluate endothelium-dependent vasodilation in the elderly: the Prospective Investigation of the Vasculature in Uppsala Seniors (PIVUS) study. *Arteriosclerosis, thrombosis, and vascular biology* **25**, 2368-2375 (2005).

20. Lawlor, D.A., Harbord, R.M., Sterne, J.A., Timpson, N. & Davey Smith, G. Mendelian randomization: using genes as instruments for making causal inferences in epidemiology. *Stat Med* **27**, 1133-1163 (2008).

21. Locke, A.E.*, et al.* Genetic studies of body mass index yield new insights for obesity biology. *Nature* **518**, 197-206 (2015).

22. Burgess, S. & Thompson, S.G. *Mendelian randomization : methods for using genetic variants in causal estimation*, (CRC Press, Taylor & Francis Group, Boca Raton, 2015).

23. Fehrmann, R.S.*, et al.* Trans-eQTLs reveal that independent genetic variants associated with a complex phenotype converge on intermediate genes, with a major role for the HLA. *PLoS Genet* **7**, e1002197 (2011).

24. Bonder, M.J.*, et al.* Genetic and epigenetic regulation of gene expression in fetal and adult human livers. *BMC Genomics* **15**, 860 (2014).

25. Yang, T.P.*, et al.* Genevar: a database and Java application for the analysis and visualization of SNP-gene associations in eQTL studies. *Bioinformatics* **26**, 2474-2476 (2010).

26. Consortium, G.T. Human genomics. The Genotype-Tissue Expression (GTEx) pilot analysis: multitissue gene regulation in humans. *Science* **348**, 648-660 (2015).

27. Grundberg, E.*, et al.* Global analysis of DNA methylation variation in adipose tissue from twins reveals links to disease-associated variants in distal regulatory elements. *American journal of human genetics* **93**, 876-890 (2013).

28. Leslie, R., O'Donnell, C.J. & Johnson, A.D. GRASP: analysis of genotype-phenotype results from 1390 genome-wide association studies and corresponding open access database. *Bioinformatics* **30**, i185-194 (2014).
