# Supplementary material for: Association of Body Mass Index with DNA Methylation and Gene Expression in Blood Cells and Relations to Cardiometabolic Disease: A Mendelian Randomization Approach
Source: PLoS Med. 2017 Jan 17;14(1):e1002215. doi: 10.1371/journal.pmed.1002215 (PMC5240936; doi:10.1371/journal.pmed.1002215)
Supplement: S1 STROBE Checklist — (DOC) [file pmed.1002215.s018.doc]

STROBE Statement—Checklist of items that should be included in reports of ***cohort studies***

|  | Item No | Completed | Recommendation |
| --- | --- | --- | --- |
| **Title and abstract** | 1 | Title describes associated study of blood methylation and expression in blood samples | (*a*) Indicate the study’s design with a commonly used term in the title or the abstract |
|  |  | Completed in abstract | (*b*) Provide in the abstract an informative and balanced summary of what was done and what was found |
|  | | | Introduction |
| Background/rationale | 2 | Completed in introduction | Explain the scientific background and rationale for the investigation being reported |
| Objectives | 3 | Stated in methods | State specific objectives, including any prespecified hypotheses |
|  | | | Methods |
| Study design | 4 | Described in methods | Present key elements of study design early in the paper |
| Setting | 5 | Described in study participants and supplemental methods | Describe the setting, locations, and relevant dates, including periods of recruitment, exposure, follow-up, and data collection |
| Participants | 6 | Described in study participants and supplemental methods | (*a*) Give the eligibility criteria, and the sources and methods of selection of participants. Describe methods of follow-up |
|  |  | N/A | (*b*) For matched studies, give matching criteria and number of exposed and unexposed |
| Variables | 7 | Described in methods and supplemental methods | Clearly define all outcomes, exposures, predictors, potential confounders, and effect modifiers. Give diagnostic criteria, if applicable |
| Data sources/ measurement | 8* | Described in methods and supplemental methods | For each variable of interest, give sources of data and details of methods of assessment (measurement). Describe comparability of assessment methods if there is more than one group |
| Bias | 9 | Described in methods and supplemental methods | Describe any efforts to address potential sources of bias |
| Study size | 10 | Described in methods | Explain how the study size was arrived at |
| Quantitative variables | 11 | Described in methods and supplemental methods | Explain how quantitative variables were handled in the analyses. If applicable, describe which groupings were chosen and why |
| Statistical methods | 12 | Described in methods and supplemental methods | (*a*) Describe all statistical methods, including those used to control for confounding |
|  |  | Described in methods and supplemental methods | (*b*) Describe any methods used to examine subgroups and interactions |
|  |  | N/A | (*c*) Explain how missing data were addressed |
|  |  | N/A | (*d*) If applicable, explain how loss to follow-up was addressed |
|  |  | Described in methods | (*e*) Describe any sensitivity analyses |
|  | | | Results |
| Participants | 13* | Described in methods and supplemental methods | (a) Report numbers of individuals at each stage of study—eg numbers potentially eligible, examined for eligibility, confirmed eligible, included in the study, completing follow-up, and analysed |
|  |  | N/A | (b) Give reasons for non-participation at each stage |
|  |  | Sample sizes included in Figure 1 | (c) Consider use of a flow diagram |
| Descriptive data | 14* | Relevant variables included in Results and Table 1 | (a) Give characteristics of study participants (eg demographic, clinical, social) and information on exposures and potential confounders |
|  |  | N/A | (b) Indicate number of participants with missing data for each variable of interest |
|  |  | N/A | (c) Summarise follow-up time (eg, average and total amount) |
| Outcome data | 15* | N/A – Single cross-sectional measure | Report numbers of outcome events or summary measures over time |
| Main results | 16 | Results | (*a*) Give unadjusted estimates and, if applicable, confounder-adjusted estimates and their precision (eg, 95% confidence interval). Make clear which confounders were adjusted for and why they were included |
|  |  | Results | (*b*) Report category boundaries when continuous variables were categorized |
|  |  | N/A | (*c*) If relevant, consider translating estimates of relative risk into absolute risk for a meaningful time period |
| Other analyses | 17 | Results and supplemental tables | Report other analyses done—eg analyses of subgroups and interactions, and sensitivity analyses |
|  | | | Discussion |
| Key results | 18 | Results | Summarise key results with reference to study objectives |
| Limitations | 19 | Discussion | Discuss limitations of the study, taking into account sources of potential bias or imprecision. Discuss both direction and magnitude of any potential bias |
| Interpretation | 20 | Discussion | Give a cautious overall interpretation of results considering objectives, limitations, multiplicity of analyses, results from similar studies, and other relevant evidence |
| Generalisability | 21 | Discussion | Discuss the generalisability (external validity) of the study results |
|  | | | Other information |
| Funding | 22 | Funding stated | Give the source of funding and the role of the funders for the present study and, if applicable, for the original study on which the present article is based |

*Give information separately for exposed and unexposed groups.

**Note:** An Explanation and Elaboration article discusses each checklist item and gives methodological background and published examples of transparent reporting. The STROBE checklist is best used in conjunction with this article (freely available on the Web sites of PLoS Medicine at http://www.plosmedicine.org/, Annals of Internal Medicine at http://www.annals.org/, and Epidemiology at http://www.epidem.com/). Information on the STROBE Initiative is available at http://www.strobe-statement.org.
