# Supplementary material for: Association of Body Mass Index with DNA Methylation and Gene Expression in Blood Cells and Relations to Cardiometabolic Disease: A Mendelian Randomization Approach
Source: PLoS Med. 2017 Jan 17;14(1):e1002215. doi: 10.1371/journal.pmed.1002215 (PMC5240936; doi:10.1371/journal.pmed.1002215)
Supplement: S1 Text — (DOCX) [file pmed.1002215.s033.docx]

**PROJECT PROPOSAL SUBMITTED TO THE FRAMINGHAM HEART STUDY PROJECT REVIEW COMMITTEE (1/31/14)**

**TITLE**: METHYLOME-WIDE ASSOCIATION STUDY (MWAS) OF BODY MASS INDEX (BMI) WITH TRANSCRIPTOMIC INTEGRATION OF TOP FINDINGS

**INTRODUCTION**

Obesity is a major global public health concern and is associated with increased morbidity and mortality. Despite the success of expansive genome-wide association studies (GWAS) in identifying novel genetic variants associated with body mass index (BMI), common genetic sequence variants only explain a modest proportion of the phenotypic variation in BMI. Genomic regulatory mechanisms that do not alter the underlying DNA sequence, such as methylation of Cytosine-phosphate-Guanine (CpG) dinucleotides, may affect BMI. DNA methylation stabilizes conformational changes in chromatin structure altering accessibility for transcriptional mechanisms. DNA methylation microarrays allow for genome-wide assays of CpG methylation at single base pair resolution in large populations. Replication of DNA methylation findings with changes in gene expression provides functional evidence supporting the significant findings. We propose a methylome-wide association study (MWAS) of BMI utilizing the Illumina HumanMethylation450 BeadChip array with interrogation of methylome-wide significant findings in gene expression from the SABRe CVD (Systems Approach to Biomarker Research in Cardiovascular Disease) project. Mediation analysis of methylation, gene expression, and BMI phenotypes will provide multilevel integration and further our understanding of how genomic regulatory mechanisms contribute to adiposity-related phenotypes. Identifying epigenetic contributions to obesity may highlight novel therapeutic targets and/or improve risk stratification for obesity-related diseases.

**STUDY AIMS**

1. To identify genome-wide differentially methylated CpGs associated with BMI and BMI-related phenotypes
2. To follow up the CpGs associated with BMI with gene expression analysis.
3. To conduct a mediation analysis of the indirect association of BMI with differentially methylated positions (identified AIM1) that may function through changes in gene expression (those replicated in AIM 2).

**METHODS**

*Study population*: We will include all Offspring cohort participants that attended exam cycle 8 (2005-2008) that have DNA methylation, gene expression, and anthropometric data available.

*Primary outcome*: 1) Body mass index (kg/m2)

*Secondary outcomes*: 2) Waist circumference (WC)

3) Waist-hip ratio (WHR)

4) Body mass index adjusted for waist-hip ratio

5) Change in BMI from exam 8 to 9, +/- adjusting for baseline BMI

6) Change in BMI from exam 1 to 8, and 7 to 8, +/- adjusted for initial BMI

*Covariates*: age, gender, estimated cell counts, technical covariates (ie. chip, etc), kinship (initial model),

Secondary models: smoking-status (current/previous/never), alcohol-intake (current / noncurrent), education (< high school, high school, >high school), physical activity index from self-report, 1-4 principal components from SNP arrays to adjust for population stratification

We will conduct additional sensitivity analyses: 1) sex-stratified, 2) excluding those with morbid obesity (BMI >= 40kg/m2 or >=35kg/m2 and obesity-related sequalae [a) dyslipidemia (Total cholesterol > 200mg/dL or LDL > 130mg/dL or HDL < 40/50 mg/dL or triglycerides > 150mg/dL) or lipid therapy, b) diabetes or on treatment for diabetes, c) hypertension (SBP >= 140 or DBP > 90mmHg) or on treatment for hypertension]

*DNA methylation:* DNA methylation data will undergo quality control measures to ensure control SNP probes on the methylation arrays match previously measured SNPs, sex extrapolated from methylation data matches expected sex, and exclusion of: samples with >1% missing rate, outliers identified by multidimensional scaling (MDS), and methylation probes with a missing rate >20%. Methylation array data will be normalized using the DASEN method implemented in the *wateRmelon* package of R (Pidsley).

*Statistical analysis*: AIM 1) Linear mixed effects regression analysis with DNA methylation specified as the dependent variable, BMI as the independent variable, and age, sex, estimated cell count as fixed effects, technical covariates as random effects and kinship as a random correlation structure using *pedigreemm* package in R. Secondary models will include the additional potential confounders as listed above as fixed effects. Sensitivity analysis will run stratified or subset analyses as described above. AIM 2) For methylome-wide significant findings (bonferroni-corrected p=0.05/480,000 ~ 1 x 10-7), we will run similar models with gene expression transcripts (gene level) from the Affymetrix arrays from genes identified in the Illumina annotation as being related to the differentially methylated CpG (upstream island/shore, intronic, etc..). For example, if a CpG in a known promoter region of FTO is associated with BMI (p<10-7), we will test the association between FTO gene transcript and BMI in similar regression models. As before, gene expression will be specified as the dependent variable. AIM 3) Mediation analyses will be conducted using multiple linear regression models first without and then adjusting for the proposed mediator. In the mediation analyses, a pathway is specified *a priori* in which a hypothesized causal factor (DNA methylation – or X in the box below) influences a mediator (gene expression – C ), which in turn affects the outcome of interest (BMI or BMI-related trait – Y ):


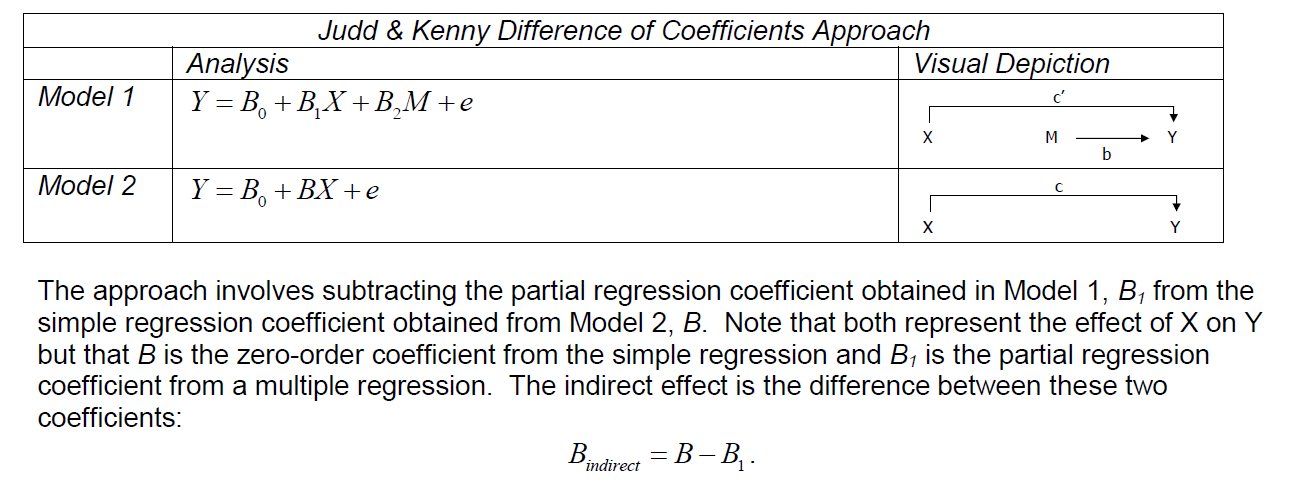


Newsom J. USP654 Data Analysis II. Portland State University

The model assumes no unmeasured confounding or effect modification between the included elements. The proportion mediated describes the average magnitude of indirect association between DNA methylation and BMI attributed through changes in gene expression relative to the average total association. Due to the expected non-normal distribution, 95% confidence intervals will be obtained from nonparametric bootstrapping with 20,000 iterations implemented in the *mediation* package in R (Imai).

*Limitations and mitigating factors*: Our analyses are limited to a single tissue type – peripheral blood cells – that may not be the tissue of primary interest for adiposity, namely adipose tissue. However, recent publications have shown that while there are regions that are differentially methylated among different tissue types (Liang), there is a very large amount of information that is shared and peripheral blood samples provide insight into trans-tissue findings. As atherosclerosis is a blood vessel disease, changes in methylation in peripheral blood samples in relation to adiposity (or our surrogate measures) are of great interest. In addition, functional analysis using ENCODE and eQTL databases will be conducted to identify regulatory regions or regions with differential expression in adipose tissue.
